# Anti-angiogenic effects of VEGF stimulation on endothelium deficient in phosphoinositide recycling

**DOI:** 10.1038/s41467-020-14956-z

**Published:** 2020-03-05

**Authors:** Amber N. Stratman, Olivia M. Farrelly, Constantinos M. Mikelis, Mayumi F. Miller, Zhiyong Wang, Van N. Pham, Andrew E. Davis, Margaret C. Burns, Sofia A. Pezoa, Daniel Castranova, Joseph J. Yano, Tina M. Kilts, George E. Davis, J. Silvio Gutkind, Brant M. Weinstein

**Affiliations:** 1 Division of Developmental Biology, National Institute of Child Health and Human Development, National Institutes of Health, Bethesda, MD 20892 USA; 2 Department of Cell Biology and Physiology, Washington University School of Medicine, St. Louis, MO 63110 USA; 3 Craniofacial and Skeletal Diseases Branch, National Institute of Dental and Craniofacial Research, National Institutes of Health, Bethesda, MD 20892 USA; 4 Department of Pharmaceutical Sciences, School of Pharmacy, Texas Tech University Health Sciences Center, Amarillo, TX 79106 USA; 5 Department of Pharmacology, UC San Diego Moores Cancer Center, La Jolla, CA 92093 USA; 6 Oral and Pharyngeal Cancer Branch, National Institute of Dental and Craniofacial Research, National Institutes of Health, Bethesda, MD 20892 USA; 7 Department of Molecular Pharmacology and Physiology, University of South Florida School of Medicine, Tampa, FL 33612 USA

**Keywords:** Tumour angiogenesis, Cell lineage, Targeted therapies

## Abstract

Anti-angiogenic therapies have generated significant interest for their potential to combat tumor growth. However, tumor overproduction of pro-angiogenic ligands can overcome these therapies, hampering success of this approach. To circumvent this problem, we target the resynthesis of phosphoinositides consumed during intracellular transduction of pro-angiogenic signals in endothelial cells (EC), thus harnessing the tumor’s own production of excess stimulatory ligands to deplete adjacent ECs of the capacity to respond to these signals. Using zebrafish and human endothelial cells in vitro, we show ECs deficient in CDP-diacylglycerol synthase 2 are uniquely sensitive to increased vascular endothelial growth factor (VEGF) stimulation due to a reduced capacity to re-synthesize phosphoinositides, including phosphatidylinositol-(4,5)-bisphosphate (PIP2), resulting in VEGF-exacerbated defects in angiogenesis and angiogenic signaling. Using murine tumor allograft models, we show that systemic or EC specific suppression of phosphoinositide recycling results in reduced tumor growth and tumor angiogenesis. Our results suggest inhibition of phosphoinositide recycling provides a useful anti-angiogenic approach.

## Abstract (web summary)

Tumors can overproduce pro-angiogenic ligands overcoming currently approved anti-angiogenic therapies and hindering their success. Here, the authors show that targeting phosphoinositide recycling during tumor angiogenesis harnesses the tumor’s own production of angiogenic ligands to deplete adjacent endothelial cells of the capacity to respond to these signals.

## Introduction

Phosphoinositides are utilized as “second messengers” for a wide variety of different intracellular signaling pathways, in virtually every cell type. This includes receptor tyrosine kinase pro-angiogenic signaling in endothelial cells (EC) through receptors like vascular endothelial growth factor (VEGF) receptor 2 (VEGFR2), the VEGFA receptor, which uses phosphatidylinositol-(4,5)-bisphosphate (PIP2) as a key substrate for both phospholipase C-gamma 1 (PLCγ1) and phosphoinositide 3-kinase (PI3K) mediated downstream signaling activation^1–11^. In order to regenerate PIP2 consumed during signaling, diacylglycerol (DAG) must be recycled to CDP-diacyglycerol (CDP-DAG) through the activity of two vertebrate CDP-diacyglycerol synthase enzymes, CDS1 and CDS2^1–8,12–17^. CDP-diacylglycerol synthase (CDS) activity is necessary to resynthesize phosphoinositide (PI), the base molecule required for the generation of downstream PI derivatives, such as PIP2. Loss or knockdown of either one of the two *CDS* genes in ECs in vitro or in zebrafish embryos in vivo results in reduced angiogenesis, while excess PIP2 promotes increased angiogenesis in wild type endothelium^16^. Importantly, the vascular defects observed in *cds2*^*y54*^ null mutant zebrafish embryos that lack Cds2 but retain Cds1 activity seem to occur in the absence of other obvious developmental abnormalities^16^, suggesting that the endothelium is differentially more sensitive to a reduced capacity to resynthesize phosphoinositides than other cell types and tissues.

Anti-angiogenic therapies have been of significant interest for a number of years due to their potential to combat tumor growth through cutting the tumor off from the host blood supply^18–24^. However, the ability of tumors to overproduce pro-angiogenic ligands and overcome targeted therapies has hampered this approach to date^25,26^. An alternative way to circumvent this problem is to target the re-synthesis of critical signaling substrates, like phosphoinositides, that are consumed during intracellular transduction of pro-angiogenic signals in ECs, thereby harnessing the tumor’s own production of excess stimulatory ligands to deplete adjacent host ECs of the capacity to respond to these signals^1,13,14,16,27–29^.

Here we show using zebrafish, human cells, and mice that ECs deficient in phosphoinositide recycling are uniquely sensitive to increased stimulation by VEGFA and other angiogenic cytokines. Instead of promoting increased angiogenesis, VEGFA stimulation of PI recycling-deficient endothelium suppresses angiogenesis and decreases pro-angiogenic signaling, suggesting highly stimulated, actively angiogenic ECs might be differentially sensitive to reduced PI recycling capacity compared to quiescent ECs. To examine whether the sensitivity of angiogenically active endothelium to reduced PI recycling capacity could be harnessed as an anti-angiogenic anti-tumor approach, we suppressed PI recycling in mice and examined the effects on growth of allografted tumors. Our results show that either systemic or EC specific suppression of PI recycling results in reduced tumor growth, reduced pro-angiogenic signaling in the endothelium, and reduced tumor angiogenesis. Together, these findings suggest that inhibition of phosphoinositide recycling may provide a useful anti-angiogenic approach, and highlights the general potential of targeting re-synthesis of rate limiting signaling substrates as a therapeutic strategy.

## Results

### Inhibiting phosphoinositide recycling sensitizes ECs to pro-angiogenic stimuli

**Fig. 1:**
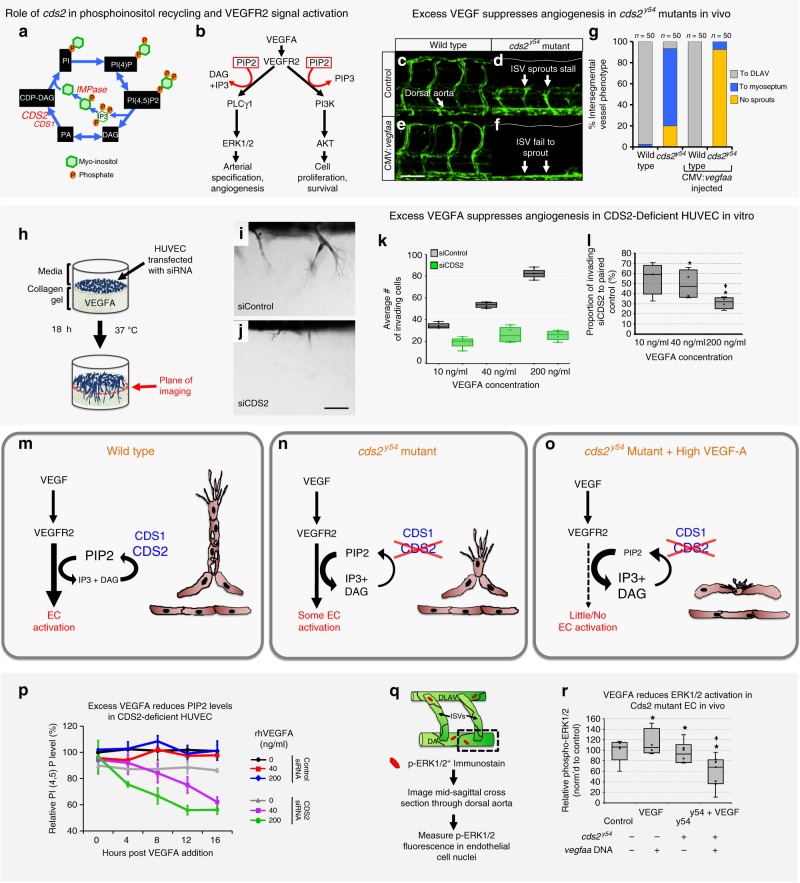
CDS2 dependent angiogenic sprouting defects in vitro and in vivo are exacerbated by exogenous VEGFA addition. **a** Schematic diagram of phosphoinositide recycling. CDS1, CDS2, and IMP enzymes facilitate regeneration of phosphoinositol after consumption of PIP2 (see Fig. S12 for details). **b** VEGFR2 signaling schematic (modified from refs. ^36,60^). **c**–**g** Confocal images (**c**–**f**) and quantitation (**g**) of trunk intersegmental vessels (ISV) in 32hpf *Tg(fli1a:egfp)*^*y1*^ WT siblings (**c**, **e**) or *cds2*^*y54*^ mutants (**d**, **f**) injected with control (**c**, **d**) or CMV*:vegfaa* (**e**, **f**) DNA. Bars in **g** measure ISV that have not sprouted (yellow), grown halfway up the trunk (blue), or formed a complete ISV (gray). Data is representative of three different experiments, *n* = 50 ISVs per treatment group. **h**–**j** HUVEC 3D invasion assay used to model angiogenesis in vitro (**h**), with representative images from control and CDS2 siRNA-treated cultures (**i**, **j**). **k** Quantification of HUVEC cellular invasion into collagen gels at VEGFA doses indicated (*n* = 4 collagen gels; data is representative of three independent experiments). **l** Quantification of CDS2 siRNA HUVEC cellular invasion normalized to VEGFA dose-matched controls (see methods); Star indicates significance from control; plus indicates significance from individual VEGFA doses (*t*-test). **m**–**o** Schematic model for phosphoinositide recycling, CDS2, and angiogenesis. **m** Under normal conditions phosphoinositide recycling maintains PIP2 levels and VEGFA signal transduction. **n** Endothelium defective for CDS2 has a reduced capacity to recycle phosphoinositides, but under conditions of initial or low-level VEGFA stimulation sufficient PIP2 is regenerated to maintain VEGFA signal transduction. **o** During sustained and/or high-level VEGFA stimulation, however, PIP2 levels cannot be maintained, leading to a collapse of VEGFA signal transduction. **p** ELISA quantitation of PIP2 levels in control or CDS2 siRNA-treated HUVECs incubated with 0, 40, or 200 ng/ml VEGFA over a 16 h time course, normalized to levels in initial control siRNA-treated HUVEC without added VEGFA. Nine technical replicates were measured per sample, per experiment. Data is graphed as the average of two experimental replicates. **q** Diagram illustrating procedure for measurement of phospho-ERK1/2 in trunk endothelial nuclei by immunofluorescence. **r** Quantitation of trunk endothelial phospho-ERK1/2 in 30 hpf *cds2*^*y54*^ mutants and WT siblings ± (with/without) CMV:*vegfaa* DNA (column 1 *n* = 5 biologically independent animals; column 2 *n* = 4 biologically independent animals; column 3 *n* = 6 biologically independent animals; column 4 *n* = 7 biologically independent animals), data is representative of two independent experiments. Star indicates significance from control; plus indicates significance from *cds2*^*y54*^ mutant—CMV:*vegfaa* DNA condition (*t*-test). Bars = 100 μm. Box plots are graphed showing the median versus the first and third quartiles of the data (the middle, top, and bottom lines of the box, respectively). The whiskers demonstrate the spread of data within 1.5x above and below the interquartile range. All data points are shown as individual dots, with outliers shown above or below the whiskers.

Previously, we reported the discovery of a zebrafish mutant in the *CDP-diacylglycerol synthase (cds2)* gene with defects in angiogenesis^16^. CDS activity is required for resynthesis of phosphoinositides (PI), including phosphatidylinositol-(4,5)-bisphosphate, or PIP2^13,14,16,30^ (Fig. 1a). In ECs, PIP2 serves as a key substrate for both phospholipase C-gamma 1 (PLCγ1) and phosphoinositide 3-kinase (PI3K) dependent signaling downstream from multiple pro-angiogenic tyrosine kinase receptors, such as VEGFR2, fibroblast growth factor receptor 1 (FGFR1) and epidermal growth factor receptor (EGFR)^1–8,31–34^ (Fig. 1b). Loss or knockdown of either one of the two *CDS* genes in ECs in vitro or in zebrafish embryos in vivo results in reduced angiogenesis (Fig. 1c, d, h–k, Supplementary Fig. 1)^16^.

From these studies, we predicted that exogenously supplying PI substrates to the vasculature would rescue the aberrant EC phenotypes in Cds2-deficient zebrafish, bypassing the need for PI recycling. Indeed, delivery of a single “bolus” intravascular injection of any PI species downstream of Cds2 dependent recycling—PI, PI(4)P, PI(4,5)P2 or PIP3—reactivates EC activity to largely reverse the angiogenic defects noted in 72 hpf *cds2*^*y54*^ mutant zebrafish embryos (Supplementary Fig. 2A–M)^16^.

To further confirm the role of PI recycling in angiogenesis, we generated a zebrafish mutant disrupting inositol monophosphatase (*impa2*^*y602*^*)* (Supplementary Fig. 3A). The *impa2* gene encodes a critical enzyme in the phosphatidylinositol recycling pathway that removes the final phosphate group from myoinositol before it is linked to CDP-DAG to regenerate PI (Fig. 1a, Supplementary Fig. 3B), a step critical for the resynthesis of all subsequent PI species. Like the *cds2*^*y54*^ mutants, zebrafish maternal *impa2*^*y602*^ mutants also display defects in trunk angiogenesis at 32 hpf (Supplementary Fig. 1C–F), demonstrating that interfering with either of two different PI recycling enzymes (Cds or Impa) impairs developmental angiogenesis. Importantly, the vascular defects observed in *cds2*^*y54*^ and *impa2*^*y602*^ mutants appear in the absence of other obvious developmental abnormalities (see ref. ^24^ for a discussion of zebrafish angiogenesis assays), suggesting that ECs are more sensitive to partial reductions in phosphoinositide recycling capacity compared to other cells and tissues. It is also worth noting that for both *cds2*^*y54*^ and *impa2*^*y602*^ there are additional isoforms of each of these enzymes that remain functional, i.e. Cds1 and Impa1, generating animals that have compromised but not completely absent PI recycling capacity.

To further explore the connections between phosphoinositide recycling and pro-angiogenic signaling, we injected a CMV*:vegfaa* transgene^35^ driving ubiquitous expression of Vegfaa into the *cds2*^*y54*^ mutant zebrafish. Instead of promoting increased vessel growth, Vegfaa overexpression in *cds2*^*y54*^ null mutants or *cds2* morpholino (MO) treated embryos results in dramatically reduced angiogenesis—including in the trunk intersegmental vessels, central arteries in the brain, and sub-intestinal vascular plexus (Fig. 1c–g, Supplementary Fig. 1). Longitudinal imaging beginning at 24 hpf reveals that vessels fail to sprout and extend properly (Supplementary Fig. 4). Sensitivity to Vegfaa is highly specific to *cds2*^*y54*^ mutants, as other zebrafish vascular mutants including *plcg1*^*y10*^*, flk1*^*y17*^, and *etsrp*^*y11*^ do not show reduced angiogenesis when injected with CMV*:vegfaa* transgene (Supplementary Fig. 5).

Human ECs in vitro also exhibit this seemingly paradoxical sensitivity to VEGFA stimulation when CDS2 activity is reduced. Two independent siRNA targets were validated in human umbilical vein endothelial cells (HUVECs), confirming that both suppressed CDS2 protein levels, disrupted HUVEC invasive capacity in vitro, and suppressed p-ERK1/2 and p-AKT signaling downstream of VEGFA stimulation (Supplementary Fig. 6A–C). Further, *CDS2* siRNA treatment reduces HUVEC proliferative capacity but does not induce apoptosis (Supplementary Fig. 6D, E). Exposing CDS2-deficient HUVECs to increasing levels of VEGFA stimulation results in decreased migratory capacity in 3D endothelial invasion assays^24^ as compared to controls (Fig. 1h–l), similar to the reduced angiogenesis noted upon VEGFA stimulation in Cds2-deficient zebrafish.

We further examined whether zebrafish vessels and HUVECs show increased sensitivity to VEGFA when inhibiting PI4K2A, PI4K2B, and PIP5K1C—the kinases that directly phosphorylate PI to generate PI(4,5)P2 (Supplementary Fig. 7)^9–11^. As with *cds2*^*y54*^ mutants, injecting exogenous CMV*:vegfaa* in zebrafish treated with PI4K inhibitor exacerbates the angiogenesis defects noted (Supplementary Fig. 7A–G). VEGF-promoted anti-angiogenic phenotypes are also recapitulated in HUVECs in which either PI4K2B or PIP5K1C are suppressed using siRNA (Supplementary Fig. 7H, I). Suppression of PI4K2B or PIP5K1C sensitizes HUVEC to exogenously supplied VEGFA, with in vitro angiogenesis reduced to a greater extent as VEGF concentration is increased (Supplementary Fig. 7H, I). Suppression of PI4K2A did not cause significant effects, nor did it cause additive effects when PI4K2A and PI4K2B were inhibited together, suggesting PIPK2B is the more critical of the two isoforms in this context.

Together, these data show that limiting PI availability via suppression of CDS2 or downstream kinases in the PI recycling pathway leads to anti-angiogenic effects that are exacerbated, not ameliorated, by increased stimulation with VEGFA.

### VEGFA stimulation results in reduced signaling in CDS2-deficient ECs

Defects in CDS2 reduces the capacity of ECs to regenerate PIs for signal transduction (Fig. 1a, b, m, n)^1,4–6,8,13,16,36,37^. We hypothesized that while initial and/or low-level VEGFA stimulation of CDS2-deficient ECs might not substantially affect PI recycling (Fig. 1m, n), higher levels and/or sustained VEGFA stimulation would gradually deplete levels of PIP2 and other phosphoinositides, halting signal transduction (Fig. 1o). To examine this directly we measured endogenous PIP2 levels over time in control and CDS2-deficient HUVECs in vitro, challenged with VEGFA (Fig. 1p). In control siRNA-treated HUVECs VEGFA stimulation itself does not alter endogenous PIP2 levels over a 16-hour time course (Fig. 1p). PIP2 levels are also not significantly reduced over 16 hours in CDS2 siRNA-treated HUVECs in the absence of exogenously added VEGFA, although the baseline level of PIP2 is slightly lower than in controls. In contrast, however, exogenous VEGFA stimulation of CDS2 siRNA-treated HUVECs results in a time-dependent decrease in PIP2 levels, with more rapid reduction in PIP2 observed at higher VEGFA concentrations (Fig. 1p). Assessing PI(4)P, PIP2 and PIP3 levels side-by-side at 12 hours after VEGFA stimulation, in lipid fractions collected from the same starting control versus CDS2-deficient HUVEC populations, reveals that all three PI species are depleted in a VEGFA dose dependent manner in roughly the same proportions (Supplementary Fig. 2N–P). Together, these data show that reducing the phosphoinositide recycling by suppressing CDS2 limits the availability of all downstream PI species.

VEGF signaling downstream from VEGFR2 and PLCγ1 is transduced via activation (phosphorylation) of ERK1/2 (Fig. 1b). Injection of a CMV*:vegfaa* transgene into control zebrafish embryos results in a modest increase in phospho-ERK1/2 levels as assessed by western blot analysis of excised zebrafish trunk tissue (Supplementary Fig. 8), or a more marked increase by immunostaining analysis examining phospho-ERK1/2 specifically in trunk ECs (Fig. 1q, r). Cds2-deficient animals show somewhat decreased phospho-ERK1/2, but injection of CMV*:vegfaa* transgene into Cds2-deficient zebrafish results in a more dramatic decrease in phospho-ERK1/2, both by western blot (Supplementary Fig. 8) and by immunostaining analysis (Fig. 1r).

The seemingly paradoxical, negative effects of increased pro-angiogenic stimulation in Cds2-deficient animals are not restricted to Vegfaa. CDS2-deficient HUVECs treated with FGF or EGF show similar reductions in signaling activation (p-AKT levels) to those seen with VEGFA treatment (Supplementary Fig. 9A–C). Furthermore, overexpressing either phospholipase C gamma (*plcγ1*) or a combination of phosphoinositol-3-kinase (*pi3k*) isoforms (both consume PIP2 during pro-angiogenic signaling; see Fig. 1b) in zebrafish *cds2*^*y54*^ mutants leads to increased inhibition of angiogenesis (Supplementary Fig. 9D–J), suggesting that inhibiting PI recycling capacity is a generalizable way to interfere with Plcγ- or Pi3k-dependent tyrosine kinase mediated signaling pathways and is not solely restricted to blocking Vegfaa signaling.

### Inhibiting phosphoinositide recycling decreases tumor growth and angiogenesis

Based on our findings, we hypothesized that phosphoinositide recycling might provide an effective target for anti-angiogenic anti-tumor treatment, as increased VEGFA and other pro-angiogenic ligands secreted by tumors in response to vascular insufficiency and hypoxia might facilitate anti-angiogenic effects, rather than help to overcome them (Fig. 1o, see summary schematic below). We used two different murine tumor allograft models, Lewis Lung Carcinoma (LLC)^38,39^ and B16-F10 (B16) melanoma^39^, to determine whether systemic or EC specific suppression of phosphoinositide recycling could specifically inhibit tumor growth and tumor angiogenesis while maintaining the normal, healthy vasculature throughout the rest of the animal. We employed five different experimental paradigms to target phosphoinositide recycling in these tumor models: direct targeting of *Cds2* using separate translation or splice blocking vivoMorpholinos (vMO)^40^, inhibition of inositol monophosphatase (IMPase) activity by the potent chemical inhibitor L-690,488^41^, inhibition of IMPase activity by lithium chloride (LiCl) treatment, and inducible, endothelial-specific genetic deletion of *Cds2* in mice via the Cre/Lox system.

**Fig. 2:**
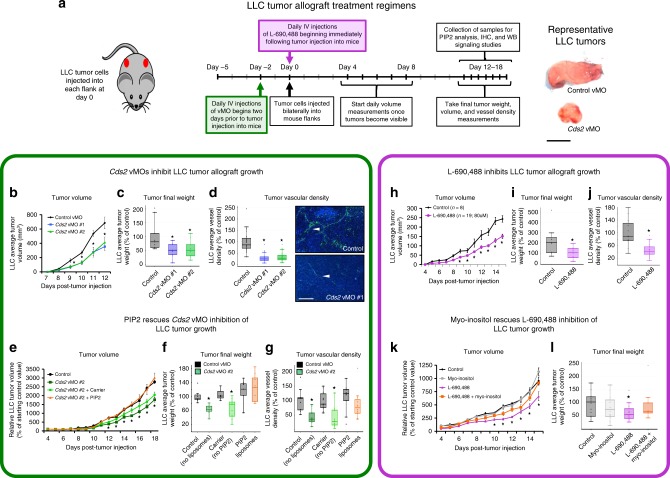
Anti-PIP2 recycling therapies suppress tumor growth. **a** Schematic of Lewis Lung Carcinoma (LLC) tumor allograft assay with representative tumor images. Bar = 1 cm. **b–g** *Cds2* vMO treatment (*n* = 10 biologically independent tumors for all groups). **h**–**l** L-690,488 small molecule inhibitor treatment (control *n* = 8 biologically independent tumors, L-690,488 *n* = 19 biologically independent tumors). Quantitation of average tumor volume (**b**, **h**), final tumor weight (**c**, **i**) and final tumor vascular density (**d**, **j**) at 12–15 days post-tumor implantation, in control versus *Cds2* vMO or L-690,488-treated animals (**b**–**d** versus **h**–**j**). **d** Quantitation of average tumor vascular density and representative images of CD31/PECAM labeled (green) versus DAPI (blue) LLC tumor sections from control vMO (top) and *Cds2* vMO (bottom) treated mice. White arrowheads indicate sites of CD31/PECAM positive blood vessel labeling. Bar = 100 μm. For all vascular density measurement experiments: three images per tumor were acquired and vascular density measured for all groups. A minimum of two slide sections from each tumor in **b**–**g** (taken from sections at least 10 slices apart) were analyzed. Representative of three experimental replicates. **e-g** PIP2 “rescues” *Cds2* vMO tumor inhibition. Quantitation of LLC average tumor volume (**e**), final tumor weight (**f**), and tumor vascular density (**g**) at 18 days post-tumor implantation in control vMO (n=8 biologically independent tumors), *Cds2* vMO #2 (no liposomes, *n* = 9 biologically independent tumors), *Cds2* vMO #2 + carrier liposome (no PIP2, *n* = 9 biologically independent tumors), or *Cds2* vMO #2 + PIP2 loaded liposome-treated (*n* = 8 biologically independent tumors), LLC-allografted mice. Data in (**e**-**g**) are normalized to the average starting size of each individual tumor group at day 4, and shown as a percentage of the starting day 4 control (the PIP2 liposome injection start date). **j** Quantitation of average tumor vascular density of LLC tumor sections from control and L-690,488-treated mice. For all vascular density measurement experiments: three images per tumor were acquired and vascular density measured for all groups. A minimum of two slide sections from each tumor in h–l (taken from sections at least 10 slices apart) were analyzed. Representative of two experimental replicates. **k**, **l** Myo-inositol “rescues” L-690,488 tumor inhibition. Quantitation of LLC average tumor volume (**k**) and final tumor weight (**l**) at 15 days post-tumor implantation in control (untreated, *n* = 14 biologically independent tumors), myo-inositol (*n* = 11 biologically independent tumors), L-690,488 (*n* = 12 biologically independent tumors), or L-690,488 + myo-inositol (*n* = 10 biologically independent tumors) treated LLC-allografted mice. Data in (**k**, **l**) are normalized to the control condition. For all panels: *p* ≤ 0.05, error bars ± SEM. Star indicates significance from control (*t*-test). Box plots are graphed showing the median versus the first and third quartiles of the data (the middle, top, and bottom lines of the box, respectively). The whiskers demonstrate the spread of data within 1.5x above and below the interquartile range. All data points are shown as individual dots, with outliers shown above or below the whiskers.

We administered murine *Cds2* translation-blocking (vMO #1) or splice-blocking (vMO #2) vMO^40^, versus a control vMO, into mice allografted with LLC (Fig. 2a) or B16-F10 (Supplementary Fig. 10A) tumors. The vMOs were introduced directly into the circulation via tail vein or retro-orbital injection to increase vascular delivery and ensure their bioavailability, beginning two days prior to the implantation of tumor cells into each flank and continuing daily throughout the time course of tumor growth (12–18 days). LLC (Fig. 2b, c) or B16-F10 (Supplementary Fig. 10B–D) allografted mice treated with either of the two *Cds2* vMOs show decreased tumor volume and decreased final tumor weight compared to control vMO-injected mice. To determine if *Cds2* vMO treatments have direct effects on tumor cell proliferation, we exposed isolated B16-F10 or LLC cells in vitro to the estimated comparable doses of *Cds2* vMO that they would encounter in vivo and measured proliferation rates. In vitro exposure of LLC or B16-F10 tumor cells to *Cds2* vMO does not alter proliferation rates, suggesting the reduced tumor growth rates we note in our in vivo vMO studies are not the result of inhibitory effects on the tumor cells themselves (Supplementary Figs. 10E and 11A). Quantitation of tumor vessel density in CD31 immunostained sections revealed ~60–80% reduction in vessel area in either LLC (Fig. 2D) or B16-F10 (Supplementary Fig. 10F–I) tumor allografts from *Cds2* vMO-treated animals compared to controls. Treatment with the vMOs does not change the overall mass of the mice (Supplementary Fig. 11B), and liver tissue from *Cds2* vMO-treated mice showed no increase in Caspase 3-positive apoptotic cells or decrease in vascularization compared to livers from control vMO-treated animals (Supplementary Fig. 11C–E).

To further address the specificity of the *Cds2* vMO treatments, we carried out “rescue” experiments using daily intravascular administration of BODIPY-PIP2 liposomes, supplied in conjunction with the retro-orbital vMO injections in LLC tumor allograft assays (Fig. 2e–g; Supplementary Fig. 11F, G; following a similar logic to that applied to the zebrafish studies in Supplementary Fig. 2). Although there are limitations associated with exogenously supplying PI substrates, including difficulties delivering lipids to desired cell types and subcellular locations, metabolism of the lipids once they enter cells, and differing fatty acid acyl chain lengths on synthetic exogenous versus endogenous phosphatidylinositides, the goal of these experiments is to bypass the defect in PI recycling in CDS2-deficient animals by directly providing a downstream PI substrate. Daily co-injection of PIP2 liposomes into LLC-allografted mice treated with the *Cds2* vMO does reverse tumor growth and vascular density defects noted with *Cds2* vMO treatment alone (Fig. 2e–g; Supplementary Fig. 11F, G). Retro-orbital intravascular injections were chosen for these studies to make the liposomes more readily accessible to the vascular endothelium compared to surrounding tissues. Indeed, immunostaining of tumor slide sections for the BODIPY fluorophore (to amplify signal), confirms enriched distribution of the PIP2 liposomes in the vasculature versus the surrounding tumor tissue (Supplementary Fig. 11H).

A similar experimental paradigm was carried out using the potent inositolmonophosphatase (IMP) small molecule inhibitor L-690,488^41^ in mice with LLC-allografted tumors. IMP catalyzes removal of the phosphate group from inositol-1-phosphate to generate myo-inositol, which is then combined with CDP-DAG (the product of the reaction catalyzed by CDS) to regenerate phosphatidylinositol- PI (Fig. 1a). LLC tumors from mice treated with L-690,488 show decreased tumor volume and final tumor weight compared to control DMSO-injected mice (Fig. 2h, i). Examination of tumor vessel density by CD31 immunostaining of sections revealed ~50–60% reduction in vessel area compared to controls (Fig. 2j). Treatment with L-690,488 does not affect the overall mass of the mice nor does it decrease normal vessel density in the liver (Supplementary Fig. 12). We carried out “rescue” experiments by co-injecting L-690,488 together with myo-inositol, the downstream product of the reaction catalyzed by IMP^16,30,41^, in order to bypass the reduced PI recycling capacity resulting from IMP inhibition (Fig. 2k, l). Myo-inositol administration effectively reverses tumor growth inhibition caused by L-690,488, suggesting the effects of this drug on tumor growth are specific to inhibition of IMP activity (Fig. 2k, l).

To further validate our L-690,488 results, we also targeted IMP activity by treating with LiCl, a compound already used in humans. Lithium is an effective inhibitor of IMP, although it also has activity against other enzymes and pathways including WNT signaling. We showed previously that LiCl inhibits angiogenesis in developing zebrafish in vivo and in EC invasion assays using HUVEC in vitro, with the specificity of these effects for IMP verified by myo-inositol rescue^16^. As with L-690,488, we find that treatment of mice with LiCl inhibits tumor growth and tumor angiogenesis in both LLC and B16-F10 allografts, and that these effects are largely reversed by co-administration of myo-inositol (Supplementary Fig. 13). To determine if LiCl treatment has direct effects on tumor cell proliferation, we exposed isolated B16-F10 or LLC cells in vitro to the estimated comparable doses of LiCl that they would encounter in vivo, and measured proliferation rates (Supplementary Fig. 13E, I). Exposure of LLC or B16-F10 tumor cells to LiCl does not alter proliferation rates at 10 and 20 uM doses, suggesting the reduced tumor growth rates we note in our study following LiCl treatment are not a result of inhibitory effects on the tumor cells themselves (as noted above and in Supplementary Figs. 10E and 11A for the *Cds2* vMOs).

**Fig. 3:**
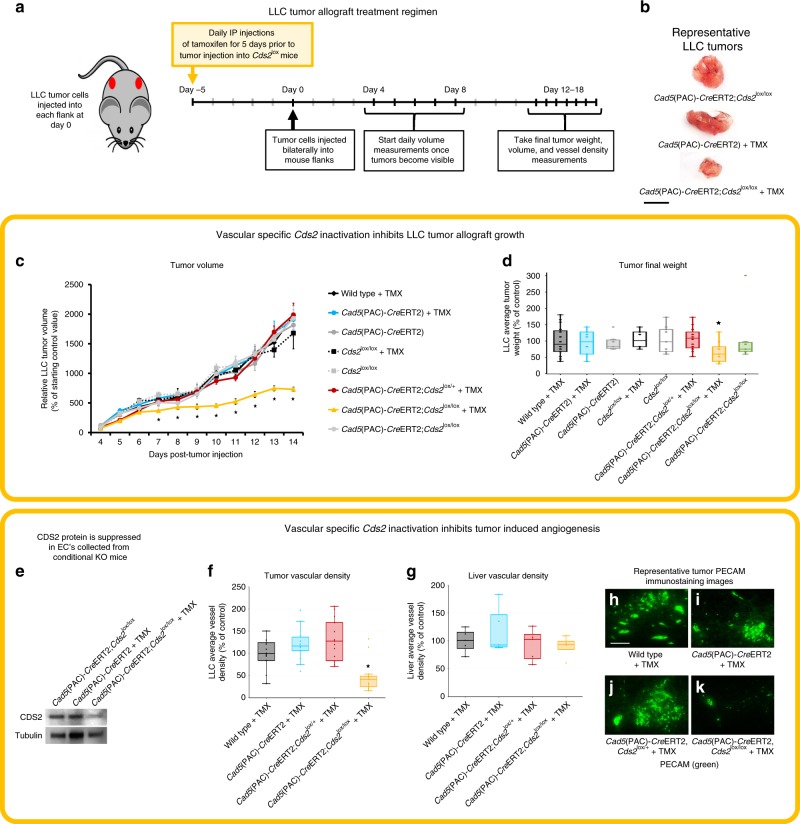
Endothelial-specific genetic deletion of *Cds2* promotes tumor growth inhibition. **a** Schematic of Lewis Lung Carcinoma (LLC) tumor allograft assay in endothelial-specific *Cds2* knockout mice. **b** Representative tumor images from Vehicle Control (*Cad5*(PAC)-*Cre*ERT2*;Cds2*^lox/lox^); Cre, TMX Control (*Cad5*(PAC)-*Cre*ERT2 +TMX); and endothelial-specific *Cds2* genetic deletion mice (*Cad5*(PAC)-*Cre*ERT2*;Cds2*^lox/lox^ +TMX). Bar = 1 cm. **c**, **d** Quantitation of LLC average tumor volume (**c**) and final tumor weight (**d**) at 14 days post- tumor implantation in the eight genetic conditions tested. Data in **c** are normalized to the starting day 4 tumor volume of Wild type + TMX controls. **e** Representative western blot images of EC protein isolated from tumors for each indicated genetic condition, probed for CDS2 and tubulin as a loading control. Blots demonstrate that CDS2 protein is still reduced in *Cad5*(PAC)-*Cre*ERT2*;Cds2*^lox/lox^ mice at day 14, after only a 5 day pulse of TMX treatment initiated prior to the start of the experiment. *p* ≤ 0.05, error bars ± SEM. *Significance from control (*t*-test). **f**, **g** Quantitation of average tumor vascular density (**f**) and average liver vascular density (**g**) of CD31/PECAM labeled LLC tumor and liver sections from control Wild type +TMX, control *Cad5*(PAC)-*Cre*ERT2 +TMX, control *Cad5*(PAC)-*Cre*ERT2*;Cds2*^lox/+^ +TMX, and endothelial-specific *Cds2* genetic deletion *Cad5*(PAC)-*Cre*ERT2*;Cds2*^lox/lox^ +TMX mice. For all vascular density measurement experiments: three images per tumor were acquired and vascular density measured for all groups. A minimum of two slide sections from each tumor (taken from sections at least 10 slices apart) were analyzed. *p* ≤ 0.05, error bars ± SEM. Star indicates significance from control (*t*-test). **h**–**k** Representative images of CD31/PECAM labeled blood vessels in tumors from control Wild type +TMX, control *Cad5*(PAC)-*Cre*ERT2 +TMX, control *Cad5*(PAC)-*Cre*ERT2*;Cds2*^lox/+^ +TMX, and endothelial-specific *Cds2* genetic deletion *Cad5*(PAC)-*Cre*ERT2*;Cds2*^lox/lox^ +TMX mice. Bar =100 μm. Naming key: Wild type + TMX (no Cre, no *Cds2* lox cassette; *n* = 18); *Cad5*(PAC)-*Cre*ERT2 +TMX (Cre^iECΔ^ only, +TMX; *n* = 13); *Cad5*(PAC)-*Cre*ERT2 (Cre^iECΔ^ only, no TMX vehicle control; *n* = 9); *Cds2*^lox/lox^ +TMX (only homozygous *Cds2* lox cassette, +TMX, no Cre; *n* = 6); *Cds2*^lox/lox^ (only homozygous *Cds2* lox cassette, no TMX vehicle control; *n* = 9); *Cad5*(PAC)-*Cre*ERT2*;Cds2*^lox/+^ +TMX (Cre^iECΔ^, *Cds2* heterozygous lox cassette, +TMX; *n* = 16); *Cad5*(PAC)-*Cre*ERT2*;Cds2*^lox/lox^ +TMX (homozygous lox cassette, Cre^iECΔ^, +TMX− experimental deletion group; *n* = 12); *Cad5*(PAC)-*Cre*ERT2*;Cds2*^lox/lox^ (homozygous lox cassette, Cre^iECΔ^, no TMX vehicle control; *n* = 7). Box plots are graphed showing the median versus the first and third quartiles of the data (the middle, top, and bottom lines of the box, respectively). The whiskers demonstrate the spread of data within 1.5x above and below the interquartile range. All data points are shown as individual dots, with outliers shown above or below the whiskers.

All of the treatments described above involve systemic inhibition of PI recycling. In order to show that the effects on tumor growth are due to targeting of the tumor-associated host endothelium rather than the tumor cells themselves, we generated inducible EC-specific *Cds2* knockout (ECKO) mice by crossing the *Cad5*(PAC)-*Cre*ERT2 EC specific Cre line^42^ to *Cds2*^tm1a(KOMP)Wtsi^ (i.e. *Cds2*^lox/lox^) mice^43^. Tamoxifen was supplied to the indicated mouse groups for 5 consecutive days prior to the implantation of LLC tumors into the flanks and then suspended for the remainder of the study. LLC tumors from the *Cad5*(PAC)-*Cre*ERT2*;Cds2*^lox/lox^ +TMX (homozygous lox cassette, Cre^iECΔ^, +TMX- experimental deletion group) show decreased tumor volume and decreased final tumor weight compared to all seven other control groups (Fig. 3a–d). Western blot data of EC’s isolated from LLC tumors from *Cad5*(PAC)-*Cre*ERT2*;Cds2*^lox/lox^ +TMX mice shows maintained, significant suppression of CDS2 protein at the end of the 14 day experimental time course (Fig. 3e). Examination of tumor vessel density by CD31 immunostaining of tumor versus liver sections revealed ~60% reduction in tumor vessel area compared to controls, with no effects on the liver vessel density (Fig. 3f–k). Importantly, for all inhibitor, MO and ECKO treatments, routine daily veterinary checks of mice in all groups did not reveal any adverse consequences, including in the ECKO group following tamoxifen treatment. *Cds2*-deleted ECKO mice ate normally and did not exhibit signs of illness throughout the course of the experiment. They also exhibited normal social interaction, grooming, and other behaviors. Some of the experimental groups implanted with tumors did become moribund and exhibit some lethargy as tumors grew large near the point of final sacrifice. This was much less evident in the ECKO tumor-implanted mice, where tumor growth was restrained compaired to the control tumor-implanted sibling groups which developed larger tumors prior to the point of sacrifice. These findings, and similar observations from our vMO, L-690,488, and LiCl experiments (Supplementary Figs. 11B, 12A, and 13N), suggest that anti-PI recycling treatments, as performed in our studies, do not result in harmful effects to the mice.

**Fig. 4:**
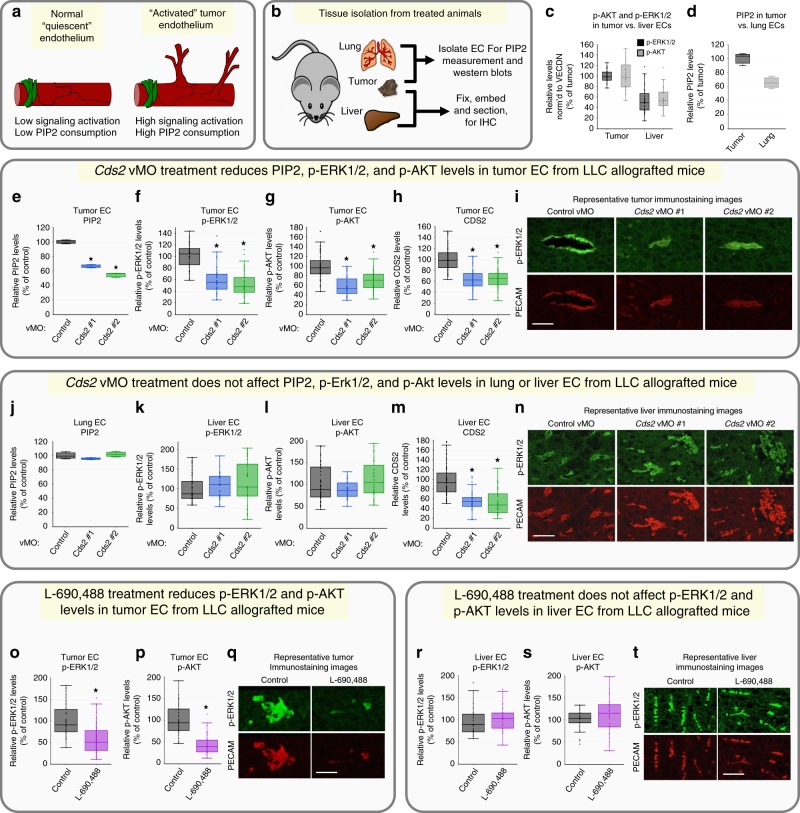
Systemic anti-PIP2 recycling therapies suppress VEGFR2 downstream signaling in LLC tumor models. **a** Model for signaling in activated versus quiescent endothelium. **b** Diagram illustrating tissues isolated from mice and their use. **c** Quantification of vascular phospho-ERK1/2 (black bars) and phospho-AKT (gray bars) levels in immunostained liver or tumor tissue from LLC-allografted control animals (*n* = 8). **d** ELISA measurement of PIP2 levels in lung or tumor endothelial cells from LLC-allografted control animals. Data is the average of three experimental replicates. **e**–**n** *Cds2* vMO-treated, LLC-allografted mice. Quantification of PIP2 levels by ELISA in tumor (**e**) or lung (**j**) endothelial cells isolated from the same LLC-allografted animals. Data per treatment condition is graphed as the average of three experimental replicates. The ELISA measurements are normalized to whole cell tubulin lysates collected at the start of the lipid isolation procedure. Quantification of vascular phospho-ERK1/2 (**f**, **k**), phospho-AKT (**g**, **l**) and CDS2 (**h**, **m**) levels from immunostained sections of tumor (**f**–**h**) or liver (**k**–**m**) tissue collected from the same LLC-allografted animals. For all immunostaining quantitation experiments: three images per individual tumor or liver were acquired and signal intensity measured for all groups. A minimum of two slide sections from each tumor or liver (taken from sections at least 10 slices apart) were utilized for independent analysis. Tumors, *n* = 10; Livers, *n* = 5. **i**, **n** Representative images of tumor (**i**) or liver (**n**) sections from control or *Cds2* vMO-treated mice, double immunostained with phospho-ERK1/2 antibody (green images) and PECAM antibody (red images). **o**–**t** L-690,488-treated, LLC-allografted mice. Quantification of vascular phospho-ERK1/2 (**o**, **r**) and phospho-AKT (**p**, **s**) levels from immunostained sections of tumor (**o**, **p**) or liver (**r**, **s**) tissue collected from the same LLC-allografted animals. **q**, **t** Representative images of tumor (**q**) or liver (**t**) sections from control (DMSO) or L-690,488-treated mice, double-immunostained with phospho-ERK1/2 antibody (green images) and PECAM antibody (red images). Control tumors, *n* = 8; L-690,488 tumors, *n* = 16; control livers, *n* = 4; L-690,488 livers, *n* = 8 p ≤  0.05, error bars ± SEM. Star indicates significance from control (*t*-test). Bars = 200 μm. Box plots are graphed showing the median versus the first and third quartiles of the data (the middle, top, and bottom lines of the box, respectively). The whiskers demonstrate the spread of data within 1.5x above and below the interquartile range. All data points are shown as individual dots, with outliers shown above or below the whiskers.

The results described above show that anti-phosphoinositide (PI) recycling treatments effectively reduce tumor growth via impaired tumor angiogenesis in LLC or B16-F10 allografted mice without significant effects on the endogenous vasculature of the liver in the same animals. We hypothesized that the increased sensitivity to anti-PI recycling treatments of tumor versus normal vessels might reflect increased signaling and increased PI substrate utilization in “activated” tumor-associated ECs compared to “quiescent” ECs in endogenous tissues and organs (Fig. 4a). We examined this in two ways: (1) by ELISA quantitation of PIP2 levels in ECs obtained from tumors and lungs collected at the termination of 14-day experiments from tumor-allografted animals, and (2) by measuring active ERK1/2 and AKT in ECs from immunostained histological sections from tumors and livers of these same mice (Fig. 4b). Tumor ECs show substantially higher phospho-ERK1/2 and phospho-AKT levels (Fig. 4c, i (upper left panel), n (upper left panel)), as well as higher PIP2 levels (Fig. 4d) compared to liver or lung ECs (respectively). The increased angiogenic signaling normally present in tumor-associated ECs, compared to ECs in endogenous host tissues, supports the idea that systemic administration of PI recycling inhibitors within an appropriate therapeutic window could result in strong effects on “activated” tumor ECs with little or no effect on normal “quiescent” ECs.

To determine whether systemic inhibition of PI recycling preferentially reduces pro-angiogenic signaling in tumor-associated ECs but not in quiescent endogenous ECs, we analyzed ECs from both tumors (Fig. 4e–i, o–q) and from livers/lungs (Fig. 4j–n, r–t) of the same *Cds2* vMO (Fig. 4e–n) or L-690,488 (Fig. 4o–t) treated, LLC-allografted mice using immunostaining and western blot analysis. Tumor-associated ECs from LLC-allografted, *Cds2* vMO-treated mice show markedly reduced levels of PIP2, p-AKT, and p-ERK1/2 (Fig. 4e–g, i; Supplementary Fig. 14G–L). Consistent with previous reports in the literature^27–29,44,45^ we find that p-AKT and p-ERK1/2 are more strongly activated in tumor-associated blood vessels than in the surrounding tumor tissue, although longer-exposure images confirm that p-AKT and p-ERK1/2 are indeed also present in the surrounding tumor cells (Supplementary Fig. 14M, N). In contrast to the tumor EC findings, EC from lungs and livers collected from the same LLC-allografted *Cds2* vMO-treated mice that the tumors were isolated from show no reduction in levels of PIP2, p-AKT, or p-ERK1/2 (Fig. 4j-l, n; Supplementary Fig. 14B–E). Importantly, tumor and liver/lung ECs from the same animals show comparable reductions in CDS2 protein levels (Fig. 4h, m; Supplementary Fig. 14A, F, G, L). Similar differential effects on tumor versus liver EC p-ERK1/2 and p-AKT activation were noted in mice treated with the IMP inhibitor L-690,488 (Fig. 4o–t).

**Fig. 5:**
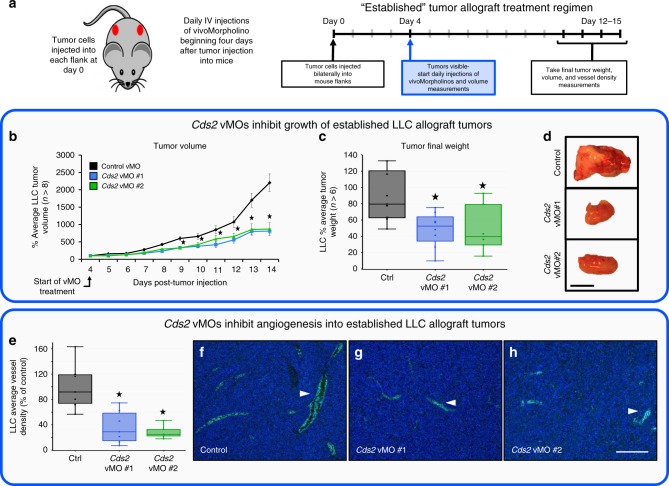
Systemic *Cds2* suppression inhibits growth of established tumors. **a** Schematic of established tumor allograft assay. Lewis Lung Carcinoma (LLC) tumor cells were injected into each flank of adult B6/C57 mice at day 0 and tumors allowed to develop for 12–15 days. Daily intravenous injections of control vivoMorpholino (vMO) or vMO targeting one of two independent sites in the *Cds2* gene (vMO #1 and vMO #2) were started at day 4 after tumor implantation. Volume measurements were taken daily starting at day 4, when tumors became visible and vMO treatment started, with final tumor volumes and weights taken at the termination of the experiment. **b** Quantitation of daily average tumor volume (in mm^3^) of LLC tumors, normalized to the average starting size of each tumor condition at day 4 (the injection start date). **c** Quantitation of average tumor weight (in mg) of LLC tumors at 14 days post-tumor implantation. Control vMO (*n* = 9); *Cds2* vMO #1 (*n* = 8); *Cds2* vMO #2 (*n* = 6). **d** Images of tumors collected from control vMO or *Cds2* vMO-treated animals at 14 days post-tumor implantation. Bar = 1 cm. **e** Quantitation of LLC tumor vessel density in control vMO- versus *Cds2* vMO-treated mice. **f**–**h** Representative images of CD31/PECAM labeled (green) LLC tumor sections from control vMO (**f**) and *Cds2* vMO (**g**, **h**) treated mice. White arrowheads indicate sites of CD31/PECAM positive blood vessel labeling. For all vascular density measurement experiments, three images per tumor were acquired (see tumor numbers above) and vascular density measured for all groups. A minimum of two slide sections from each tumor (taken from sections at least 10 slices apart) were utilized for independent analysis. Bar = 100 μm. Box plots are graphed showing the median versus the first and third quartiles of the data (the middle, top, and bottom lines of the box, respectively). The whiskers demonstrate the spread of data within 1.5x above and below the interquartile range. All data points are shown as individual dots, with outliers shown above or below the whiskers.

Finally, to determine whether systemic inhibition of PI recycling can slow the growth of pre-existing tumors, we allografted LLC tumors into mice and allowed the tumors to grow and establish. Mice were then administered *Cds2* translation-blocking (vMO #1) or splice-blocking (vMO #2) vMO, versus a control vMO daily through retro-orbital injections (Fig. 5a). LLC-allografted mice treated with either of the two *Cds2* vMOs show decreased tumor volume and decreased final tumor weight compared to control vMO-injected mice (Fig. 5b–d). Quantitation of tumor vessel density in CD31 immunostained sections revealed an ~70–80% reduction in vessel area in LLC tumor allografts from either of the *Cds2* vMO-treated animals compared to controls (Fig. 5e–h).

To determine whether systemic inhibition of PI recycling still preferentially reduces signaling in tumor-associated ECs as compared to quiescent endogenous ECs in this model of pre-exisiting tumors, we analyzed ECs from lungs (Supplementary Fig. 15A–E) or tumors (Supplementary Fig. 15F–J) of the same *Cds2* vMO treated, LLC-allografted mice via Western blot analysis. ECs collected from the tumors have reduced PIP2, p-AKT, p-ERK1/2, and CDS2 protein levels following *Cds2* vMO treatment as compared to control treatments (Supplementary Fig. 15F–J), while EC from lungs of the same *Cds2* vMO-treated mice show minimal or no reduction in PIP2, p-AKT, or p-ERK1/2 levels (Supplementary Fig. 15A–E), despite comparable reduction in CDS2 protein levels.

Taken together, these data show that systemic inhibition of PI recycling causes angiogenic defects in the tumor-associated vasculature, but has little effect on the pre-existing stable lung or liver vasculature of the same animals.

## Discussion

In this study we show that reduced capacity of ECs to recycle PI substrates results in specific defects in angiogenesis and VEGF-mediated signal transduction that correlate with reduced levels of PIP2 in highly active EC populations, such as the tumor vasculature. Zebrafish lacking CDP-diacyglycerol synthase 2 (Cds2), one of two enzymes required for re-synthesis of CDP-diacylglycerol (CDP-DAG) from diacylglycerol (DAG), show specific defects in sprouting and growth of angiogenic vessels, while appearing otherwise normal through early larval stages of development^16^. Zebrafish with maternal depletion of the Impase, the enzyme required for the re-sysnthesis of myo-inositol, show this same defect. These results suggest that the highly active angiogenic ECs present in developing vascular networks may be utilizing phosphoinositide signaling and consuming PI substrates to a greater extent than most other cell types.

**Fig. 6:**
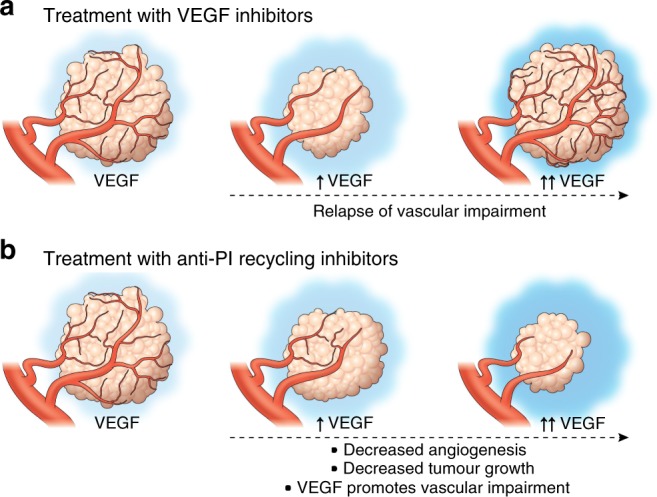
Proposed model for inhibition of tumor growth with anti-VEGF versus anti-PI recycling therapies. **a** Currently available anti-angiogenic therapies targeting VEGF lead to an initial decrease in tumor growth and angiogenesis. However, production of high levels of pro-angiogenic ligands by tumors results in a relapse in vascular impairment and return of tumor growth. **b** Our results indicate that targeting PI recycling leads to reduced tumor vasculature and tumor growth, and further suggest that the effects on tumor angiogenesis and tumor growth may be strengthened, not overcome, as tumor production of VEGFA and/or other cytokines increases.

We show that ECs are not only sensitive to reduced capacity to re-synthesize phosphoinositides, but that this sensitivity is exacerbated by increasing VEGFA stimulation (Fig. 1, Supplementary Fig. 1). VEGFA has a well-documented role as a key pro-angiogenic ligand- increased VEGFA stimulation typically leads to increased angiogenic sprouting and growth of vessels. Our results show, however, that VEGFA stimulation of ECs with compromised capacity to recycle phosphoinositides has the seemingly paradoxical opposite effect, resulting in less VEGFA-dependent signaling and less vessel growth. This suggests that targeting phosphoinositide recycling might prove to be a useful approach for anti-angiogenic cancer therapy, as tumors increasing VEGFA production in response to oxygen or nutrient deprivation resulting from vascular insufficiency caused by inhibition of tumor endothelial PI recycling might actually increase, rather than decrease, the effectiveness of these types of inhibitors (Fig. 6). We demonstrate here that inhibiting phosphoinositide recycling in vivo does result in reduced tumor growth, reduced tumor angiogenesis, reduced angiogenic signaling, and reduced PIP2 levels in the tumor-associated vasculature in multiple murine tumor allograft models without apparent effects on the normal, quiescent vasculature of the same animals (Figs. 2–5 and Supplementary Figs. 10–15).

We would note again that *CDS2* and *IMP* knockdown and the other treatments we employ in this study only result in a partial loss of phosphoinositide recycling capacity. Our data suggests that in normal “resting” tissues, or even in moderately angiogenically active vessels, the residual capacity to recycle PIs by secondary CDS or IMP enzymes is adequate to regenerate sufficient PI substrates to maintain baseline intracellular signaling (Fig. 4 and Supplementary Figs. 14, 15). On the other hand, under conditions of high angiogenic stimulation, such as in B16-F10 or LLC tumor-associated vessels or in highly angiogenically active developing vessels in the zebrafish, vascular defects appear (Figs. 1 and 4, and Supplementary Figs. 1, 3, 5). Although we did not observe any obvious negative effects of our anti-PI recycling treatments in mice, it remains to be seen whether longer term anti-PI recycling treatments might eventually lead to some effects on angiogenically active normal tissues. However, some hints to the long-term sustainability and effectiveness of anti-PI recycling therapies may possibly be gleaned from the historically well-tolerated and continued use of LiCl for management of bipolar disorder and cognitive impairment in human patients^30,46–48^.

In our work, we demonstrate anti-angiogenic, anti-tumor effects of LiCl and L-690,488, two chemical inhibitors that target the IMPase enzyme to prevent PI/PIP2 regeneration (Figs. 2 and 4, and Supplementary Fig. 13). Myo-inositol, the downstream product of IMPase that both chemicals block the production of^30^, is able to significantly rescue the inhibitory effects elicited by these drugs (Figs. 2 and 4, and Supplementary Fig. 13). This rescue suggests that many of the anti-angiogenic, anti-tumorigenic effects we note in our inhibitor treatments are directly related to PI recycling and not off-target effects, though the exact mechanism requires further investigation. Since lithium is already used in the management of bipolar disorder^30^, it would be of interest to determine whether patients treated with lithium display a reduced risk of cancer or cancer related morbidity compared to the general population. At present, the epidemiologic data related to cancer incidence are inconclusive, likely due in part to small sample sizes in the studies carried out and the complexity of the populations being evaluated, with some studies reporting reduced rates of cancer mortality and morbidity^49,50^ and others not reporting such an association^51–53^. Additional, carefully designed studies using large patient populations will be needed to better examine whether there is a demonstrable link between cancer risk and lithium use. However, as noted above, the historic use of lithium in patient populations suggests that at therapeutically appropriate doses, treatment with IMPase inhibitors can be well-tolerated over time.

In conclusion, our findings highlight the role that phosphoinositide recycling plays in tumor angiogenesis, and suggest that targeted inhibition of PI recycling may provide a useful anti-angiogenic modality for the treatment of cancer (Fig. 6). The seemingly paradoxical sensitivity of ECs subjected to anti-PI recycling treatments suggests that high levels of tumor-secreted, pro-angiogenic cytokines may facilitate the effectiveness of anti-PI recycling therapies rather than overcome them (Fig. 6). Our data also raises the possibility that anti-PI recycling therapy might not only inhibit tumor angiogenesis, but be even more effective against aggressive tumors overexpressing high levels of VEGFA and other pro-angiogenic cytokines that have traditionally been the most refractory to treatment^25,26^. Additional studies will be needed to determine whether anti-PI recycling does indeed provide a useful therapeutic modality for treatment of these aggressive cancers.

## Methods

### Zebrafish methods

Zebrafish (Danio rerio) embryos were raised and maintained as described^54,55^. The *Tg(fli1a:EGFP)*^*y1*^ transgenic zebrafish line was previously described^2^. The *cds2*^*y25*^ and *cds2*^*y54*^ mutants were identified in an F3 genetic screen in the *Tg(fli1a:EGFP)*^*y1*^ background as described previously^16^. The *impa2* ^*y602*^ mutant in the *Tg(fli1a:eGFP)*^*y1*^ background was newly generated for this manuscript. Embryos imaged at developmental stages later than 36 hpf were treated with 1-phenyl-2-thiourea to inhibit pigment formation^55^. Zebrafish husbandry and research protocols were reviewed and approved by the NICHD Animal Care and Use Committee.

### Genotyping assays for *cds2*^*y25*^ and *cds2*^*y54*^ mutants

*cds2*^*y25*^: Zebrafish carrying the *cds2*^*y25*^ mutation were genotyped using KBiosciences Competitive Allele-Specific PCR genotyping system (KASP) assays. Assays were performed on zebrafish adult or embryonic genomic DNA extracts. Primer mixes with two 5′ fluor-labeled oligos that bind to allele-specific primers are provided with a common 3′ oligo, as per the manufactures design. y25FAM, 5′-CAGCGGGAGGAGCC TCTTC-3′ and y25HEX- 5′-GCAGCGGGAGGAGCCTCTTT-3′; y25common, 5′-AAATGA AGCGATGGTATTTGCTGAGGATT-3′. Details on assay and primer design can be obtained from KBioscience. The following PCR program was designed to determine wild-type versus mutant PCR fragments: (1) 94 °C, 15 min; (2) 94 °C, 20 s; (3) 61 °C 1 min; (4) GOTO step 2 9x and decrease temperature in step 3 by 0.6 °C each cycle; (5) 94 °C, 10 s; (6) 55 °C, 1 min; (7) GOTO step 5 35x; (8) 25 ^°^C, 5 min; Plate Read and End. As primers are labeled with fluorophores, homozygous products express only one fluorescent signal while heterozygous products will result in a mixed fluorescent signal.

*cds2*^*y54*^: PCR was performed on zebrafish adult or embryonic genomic DNA extracts using REDTaq ReadyMix PCR Reaction Mix and the following primers: y54genotypeF- 5′-AACAGCTTGATGTAGCACAGCAGAGTA-3′; y54genotypeR- 5′-ATGAGGGTGTGGATGATGATGATA-3′. PCR products were then digested with MseI cutting the PCR products carrying mutant *cds2* into 193 and 124 bp fragments versus wild-type *cds2* products that do not cut generating a 317 bp PCR product. All fish were housed as pairs in genotyping tanks (R&D Aquatics).

### Zebrafish expression constructs, CRISPR and morpholinos

CMV*:vegfaa-* pCS2(+)-*zvegf*_*165*_ was a gift from Dr. Ruowen Ge (National University of Singapore). CMV promoter-driven zebrafish *vegf*_*165*_ DNA for injection was generated as previously described. DNA was injected at a final concentration of 75 ng/μL. Zebrafish pCR-*plcg1* was MluI digested and pCSDest-*pik3cd* or *pik3c2b* was ClaI digested for 1 hour prior to DNA injection at the single cell stage at 75–100 ng/μL.

Morpholino antisense oligonucleotides (Genetools) used in this study include:

*cds2* ATG MO: 5′-TCGCTGTCGTAATTCTGTCATGGTG-3′ that targets −4 to 21 of the 5′ untranslated region and coding region of *cds2* (1.8 ng was used as a maximal dose);

*vegfaa*: 5′-CTCGTCTTATTTCCGTGACTGTTTT-3′ (6 ng was used as a maximal dose). The *cds2* and *vegfaa* morpholinos used have both been previously validated^16,56^. Morpholino injections were performed on zebrafish embryos in the 1– cell stage as previously described in ref. ^2^. Maximal doses were determined by titration of morpholinos to levels that yielded maximal vascular-specific phenotypes with little to no nonspecific toxic effects.

PI4K inhibitor (PIK-93, #B-0306, Echelon Biosciences) was resuspended in DMSO and used on the zebrafish at 20-40 uM.

The *impa2* ^*y602*^ mutant allele was generated using the CRISPR/Cas9 system. The following guide RNAs were transcribed in vitro using the MEGAscript T7 Kit (Invitrogen), and injected at a dose of 150 pg/nl per embryo:

TAATACGACTCACTATAGGGCGTCAGGTTTATTGGTGGTTTTAGAGCTAGAA.

pT3TS-nCas9 (Addgene) was transcribed using MEGAscript T7 Kit (Invitrogen), and injected at a dose of 300 pg/nl per embryo. Embryos were injected at the single cell stage, screened for cutting efficiency and grown on system. F1 generations were analyzed for mutations, and pairs crossed for analysis in the F2 and beyond generations.

ABI 3130xl Fragment Analyzer Protocol for Genotyping *impa2* ^*y602*^ mutants.

PCR protocol with AmpliTaq Gold DNA Polymerase 1×(10 μl) Rxn: 1 μl 10x PCR Gold Buffer; 0.5μl MgCl_2_ 25 mM; 1 μl 0.5 mM ABI Fwd primer; 1 μl 1 mM ABI Rev primer; 0.2 μl 10 mM FAM-M13 primer; 0.1 μl dNTP Master Mix; 0.1 μl TaqGold polymerase; 1 μl of 1:10 diluted crude gDNA; 5.1ul H2O.

TaqGold PCR Program: 95 °C 10 min; 95 °C 30 s; 58 °C 30 s; 72 °C 30 s (1 min/kb); GoTo Step 2 x34; 72 °C 10 min; 15 °C Hold; Run on ABI immediately or store at 4 °C in the dark for 24 h max.

ABI 3130xl Pate set-up: HiDi Formamide/ROX master mix- 0.2 μl ROX300HD; 9.8 μl HiDi Formamide; add 10 μl of master mix to each ABI plate sample well; add 2 μl of fluorescent PCR product; cap wells and denature at 95 °C for 5 min; uncap all wells and replace with ABI plate septa to run on the 3130xl. Follow manufacturer directions to utilize the ABI 3130xl.

Gene Specific primers for genotyping:

*impa2:*

FW-M13: TGTAAAACGACGGCCAGTCTTACTGTGACATGTTAATGTGATG

RV-PIG Tail: GTGTCTTGCACAAAGTTACAGGTGCCGTCGATG

FAM-M13: 5′-/56-FAM/ TGTAAAACGACGGCCAGT-3′.

### siRNA transfection and validation

Invitrogen single SilencerSelect or Stealth siRNAs for CDS2 versus a siRNA negative control (control) were purchased and resuspended in H_2_O at a concentration of 10 μM. siPORT Amine (ThermoFisher #AM4503) was used as a transfection agent following manufacturer recommendation (siRNA target sequences in Supplemental Table 1). A double transfection protocol, with a day of “rest” between treatments, was used as previously described^6,57^ with a final concentration of 50 nM siRNA per transfection condition. Validation of CDS2 siRNA suppression was previously reported^16^ but reconfirmed here by western blot. The siRNA sequences utilized are as follows:

CDS2 #1 (Stealth): GAGUACAACAAUGACACCAACAGCU

CDS2 #2 (Silencer Select): GUUUUAUCAGAGGCCCUAAUU

PIP5K1C (Silencer Select- Validated): GCGUCGUGGUCAUGAACAAtt

PI4K2A (Silencer Select- Validated): CCAAAGAUAUCGGACCCUAtt

PI4K2B (Silencer Select- Validated): GAUUGACCGUGCAAAAUCAtt.

### Endothelial cell culture and assays

HUVECs (Lonza) were cultured in bovine hypothalamus extract, 0.01% Heparin and 20% FBS in M199 base media (Gibco) on 1 mg/mL gelatin-coated tissue culture flasks. HUVECs were used from passages 3–6.

3-dimensional (3D) in vitro angiogenesis assays were done in 2.5 mg/mL collagen type I (BD Bioscience, Acid Extracted) gels, prepared including SCF (R&D Systems, #255-SC/CF), SDF1α (R&D Systems, #350-NS/CF) and IL-3 (R&D Systems, #203-IL/CF in the gel at 200 ng/mL and VEGFA (R&D Systems, #293-VE/CF) at the indicated doses described in the manuscript, essentially as described^6,57^. HUVECs were seeded on the gel surface at a density of 40,000 cells/well. Culture media included FGF (200 ng/mL; R&D Systems, #233-FB-025/CF), IGF-II (200 ng/mL; R&D Systems, #292-G2-250), and ascorbic acid (Sigma). Assays were fixed in 3% glutaraldehyde at the indicated time points and processed for future analysis. Cells were stained with 1% toluidine blue in 30% methanol to increase visualization before imaging. Data analysis was done by imaging the endothelial cell invasive front at 3 depths below the monolayer of cells (~50 μM apart in depth). Images were obtained and the number of endothelial cells present at each depth counted; the counts at the 3 depths were added together to give a total number of invading cells per well. A minimum of five replicate wells was averaged to give a mean ± SD within a single experiment. At least 2 experimental replicates were performed. Data is represented as a percentage of the 10 ng/ml VEGFA Control siRNA condition (Fig. 1j- % Average number of invading cells) or as a percentage of invasion inhibition at each indicated VEGFA dose (Fig. 1h- calculated by taking % Invading number of CDS2 KD cells/% Invading number of Paired Control cells = % Invasion Inhibition).

For signaling activation assays, endothelial cells were seeded as a confluent monolayer for 24 h in full growth media. The morning of the assay, cells were serum starved in basal 1x M199 media for 4 h. The indicated growth factors/ligands were then supplied at a dose of 40 ng/ml in the basal media for 10 min. Lysates were collected and analyzed following the methods outlined below.

### PI isoforms quantification

siRNA-treated HUVECs were plated on collagen type I-coated plastic wells, allowed to attach overnight and then treated with the indicated doses of rhVEGFA for predetermined times prior to PI(4)P, PI(4,5)P and PIP3 quantification. Quantification was done using a competitive ELISA Kits purchased from Echelon Biosciences (Echelon Biosciences,#K-4000E, #K-4500, #K-2500 s) or utilizing a direct ELISA protocol for measuring PIP2 levels as previously modified and described^58^. Lipids was extracted following Echelon Biosciences manufacturer recommendations. Absorbance was read using a plate reader at 450 nm. No detergent was used for washes to maintain the PIP2 lipid integrity. Samples were assessed in triplicate, normalized to cell number as assessed by counting four individual high powered fields (trypsin treatment of the cells can alter lipid levels and is not advised if it can be avoided), and results given as mean relative PIP2 levels as compared to control untreated cells, error bars ± SD for a single experiment or error bars ± SEM when averaging multiple experiments. At least 2–3 experimental replicates were performed.

For measurements of PIP2 levels in lung ECs and tumor-associated ECs, competitive ELISAs protocols were done as described above. Results were normalized to input protein levels based on Western blot tubulin expression.

### Liposome injections in Zebrafish and mice

Purified PI(4,5)P2 liposomes (Echelon Biosciences, #P-9045; to note- the SN2 position is 6-carbons long; the SN1 position, where the BODIPY is attached, is 6-carbons long and joined with an amide linker) were incubated in equal molar concentration (40 μM) with lipid carrier constructs (Histone H1 Carrier #2, Echelon Biosciences) for 10 min at room temperature prior to intra-vascular injection into either the zebrafish or mice (via retro-orbital injection). For zebrafish studies, a single 15 nl “bolus” injection of BODIPY labeled PIP2 was delivered over the course of 2–3 min into the sinus venosus at 48 hpf. Only fish showing substantial and predominantly vascular dispersion of the BODIPY labeled PIP2 were utilized for ISV quantification analysis. Data analysis was done at 72 hpf. This same protocol was followed for PI (#C-00M6, Echelon Biosciences), PI(4)P (#C-04M6, Echelon Biosciences), and PIP3 (#C-39M6, Echelon Biosciences) liposome injections. For mouse PI(4,5)P injection studies, daily retro-orbital injections of BODIPY PIP2 liposomes were done (40 μM) versus lipid carrier (Histone H1 Carrier #2, Echelon Biosciences) alone conditions.

### Immunoanalysis and immunostaining

Immunoanalysis: Zebrafish trunk tissue was collected using no. In all, 55 superfine forceps to remove the head and dissect off the yolk ball. Tissue was directly lysed in 2x Laemmli Sample Buffer containing 5% β-ME and a PhosSTOP tablet (Roche), 10 ul per embryo. HUVEC culture lysates were harvested directly into the same lysis buffer described above. Antibodies: p-ERK1/2 (#4370), T. ERK (#4695), p-AKT (#4060), T. AKT (#2920) (Cell Signaling Technologies, all 1:1,000), Tubulin (Sigma; #T6199- 1:10,000), CDS2 (ProteinTech, #13175-1-AP-1:500), BODIPY (Invitrogen, #A5770- 1:1000) in 5% BSA. Secondary HRP-conjugated antibodies were purchased from Santa Cruz or Invitrogen and used at 1:2000 in 5% milk. Quantification of relative band intensity was performed by ImageJ image analysis software, with results shown from at least two independent HUVEC assays, two independent zebrafish clutches, or two-three independent western blots from pooled EC samples collected from a minimum of 2-4 tumors/lungs. Data is reported as a percentage of control levels, and p-ERK1/2 and p-AKT are normalized to total tubulin levels.

Immunostaining: Zebrafish and 3D collagen assays utilized for immunostaining analysis were fixed in 4% or 2% PFA, respectively, at 4 °C overnight. Both were immunostained following the same basic protocol: (1) 30 min RT incubation in Tris-Glycine; (2) 1 h RT incubation for permeabilization with 0.01% TritonX-100; (3) 2 h RT incubation in blocking solution (5% Sheep Serum, 1% Roche Blocking Buffer in PBST); (4) 1 h at RT or overnight 4 °C incubation with 1:1000 primary antibody; (5) wash with PBST; (6) 2–3 h RT incubation with 1:2000 AlexaFlour secondary antibody in 5% Sheep Serum, 1% Roche Blocking Buffer in PBST; (7) wash with PBST and imaging analysis. For mouse tumor and tissue slide sections: the same essential staining protocol was utilized following (1) incubation of the slides at 50 °C to melt the paraffin wax, (2) 3x, 10 min wash in Histoclear (xylene alternative), (3) 2x, 5 min wash 100% EtOH, (4) 5 min was, 90% EtOH, (5) 5 min wash 70% EtOH, (6) continue to above staining procedure. All tissues were mounted and sectioned using the services of Hisoserv, Inc, Germantown, Maryland. All slides stained for p-AKT and p-ERK1/2 were co-stained with PECAM/CD31 antibody to confirm vascular expression for quantification purposes.

Quantification of immunostaining intensity was performed by ImageJ analysis software. Images were acquired using a Leica SP5 II confocal microscope. All images were acquired at the same intensity, step size and image resolution for analysis. Data is reported as the percent average intensity per region of interest size. For all vascular density measurement and pERK1/2/pAKT fluorescent quantitation experiments three images per tumor were acquired (see tumor numbers in the figure legends) and average measurements taken for all groups. A minimum of two slide sections from each tumor (taken from sections at least 10 slices apart) were utilized for analysis, data ± s.e.m. Vascular expression of p-AKT and p-ERK1/2 was in every case confirmed by co-staining with PECAM/CD31 antibody.

### Tumor allografts

All animal studies were carried out according to NIH-approved protocols, in compliance with the Guide for the Care and use of Laboratory Animals. B16-F10 or LLC tumor cells were maintained in 10% FBS/DMEM growth media and obtained from the JS Gutkind lab. Prior to tumor injection into B6/C57 mice, the mice were shaved to remove hair at the site of tumor growth and facilitate tumor identification and volume measurements. On the day of injection, the tumor cells were trypsinized and resuspended in PBS. In all, 200 μl of PBS/cell suspension containing 10^6^ cells was injected into each flank. For LiCl studies, normal saline (control), LiCl (400 mg/kg), myoinositol (400 mg/kg), or LiCl+myoinositol in 200 μL normal saline were injected daily IP. For vMO (Gene Tools) studies, control vMO (sequence: CCCTGTCCCCTACTTCGCTCATGCT), *Cds2* vMO #1 (CCTCTGCCG TAGTTCGGTCATGCT), *Cds2* vMO #2 (GGCTTGTCAACGATACTTACAATCA) were injected through the tail vein or retro-orbitally at a dose of 40 μl of 2000 nmol stock per mouse per day. vMO were stored at room temperature and heated at 65 °C for 15 min prior to use. To verify suppression of the CDS2 gene in vMO-treated mice, endothelial cells were isolated from the lungs at the termination of the studies (see below), and lysed for protein analysis to verify protein suppression. L-690,488 chemical inhibitor was purchased from Tocris (#0682), resuspended in DMSO at 100 mM and supplied via retro-orbital injection at a daily dose of 80uM per animal daily. For endothelial-specific genetic suppression of *Cds2*, tamoxifen was injected IP daily at a dose of 1 mg/mouse in corn oil for 5 days prior to allograft of the tumors. The *Cds2* mouse strain used for this research project was created from ES cell clone EPD0033_1_C08 obtained from KOMP Repository (www.komp.org) and generated by the Wellcome Trust Sanger Institute, and genotyped following their explicit instructions^43^. The *Cad5*(PAC)-*Cre*ERT2 were used with permission from Dr. Ralf Adams, and genotyped following their experimental protocols^42^. Endothelial cells were isolated from both lungs and tumors at the end of the 14 day experiment to confirm Cds2 protein suppression. Eight groups were generated:

Wild type + TMX (no Cre, no floxed *Cds2*, tamoxifen added CONTROL)

*Cad5*(PAC)-*Cre*ERT2 +TMX (cre driver alone, tamoxifen added CONTROL)

*Cad5*(PAC)-*Cre*ERT2 (cre driver alone, no tamoxifen CONTROL)

*Cds2*^lox/lox^ +TMX (homozygous floxed *Cds2*, no cre, tamoxifen added CONTROL)

*Cds2*^lox/lox^ (homozygous floxed *Cds2*, no cre, no tamoxifen CONTROL)

*Cad5*(PAC)-*Cre*ERT2*;Cds2*^lox/+^ +TMX (heterozygous EC-specific knockout)

*Cad5*(PAC)-*Cre*ERT2*;Cds2*^lox/lox^ +TMX (homozygous EC-specific knockout)

*Cad5*(PAC)-*Cre*ERT2*;Cds2*^lox/lox^ (cre driver, homozygous floxed *Cds2*, no tamoxifen CONTROL).

For all experiments, calipers were used to measure the approximate tumor volume daily, with final weight measurements (mg) made at the termination of the experiment. Tumors were allowed to grow until control tumors reached a volume of ~500–1000 mm^3^, day 18 was reached, or significant impairment to the mouse motility from tumor burden was noted.

### Measurement of tumor vascularization

B16-F10 or LLC tumors or livers from the treatment mice were collected for analysis and paraffin embedded for H&E and immunofluorescent staining or preserved in OCT medium for cryo-sections. At minimum, four livers or eight tumors of each type were embedded and serially sectioned for vessel density analysis. Immunofluorescent labeling of CD31/PECAM (BD Biosciences #550274, 1:10) and active Caspase3 (Sigma, #C4748- 1:1000) was done and slides counterstained with HOECHST (Molecular Probes #33342, 1:2000). Images were taken using a Leica Inverted TCS-SP5 II confocal microscope and 20x objective. Three images were taken randomly from within the liver or the tumor body and the average vessel area measured per high powered imaging field. Average vessel area was measured using ImageJ, measuring the average CD31/PECAM+ area per high power image field. The data are reported as a mean, error bars ± SEM.

### Quantification of tumor cell proliferation

For tumor cell proliferation in the presence of LiCl: B16-F10 or LLC tumor cells were plated in 24-well plates in 500 μl of 10% FBS/DMEM. Cells were allowed to attach and LiCl or vMO added. Estimated concentrations of LiCl were determined proportionally based off of doses provided in vivo to the mice (whole mouse dose/approximate tissue volume = x concentration). In all, 10 μM (estimated equivalent concentration to mouse dose) and 20 μM (estimated double concentration to mouse dose) concentrations of LiCl were added to the growth media daily and proliferation assessed over a multi-day time course. Culture media was changed completely every 3 days for the duration of the time course. For tumor cell proliferation in the presence of vMO: Concentrations for the vMOs were determined based off of the estimated mouse blood volume (~2 ml) and proportioned to the volume of tissue culture media present in the assay. A daily dose of 4 μl of the 2000 nmol stock per well was added to 24-well plates containing 500 μl of 10% FBS/DMEM. Media was changed completely every 3 days. For both assays, cells were fixed using 2% PFA and stained with HOECHST dye. Five distinct images per well were assessed and the number of nuclei per well counted. The data are reported as the averages between images ± SD.

### Isolation of mouse lung and tumor-associated endothelial cells

Isolation of mouse lung or tumor microvascular endothelial cells was performed essentially according to a previously described protocol^59^. Modifications are briefly described, lungs were removed from the mice, washed in 10% FBS/DMEM, minced into 1–2 mm squares and digested with Collagenase Type I (2 mg/ml, Gibco) at 37 °C for 2 h with occasional agitation. The cellular digest was filtered through a 70 μm cell strainer, centrifuged at 1.2 g and the cells immediately incubated with Dynabead sheep anti-rat IgG (Invitrogen) coated with a rat anti-mouse ICAM-2 mAb (3C4, 22.5 μL of antibody per 150 ul of beads) at room temperature for 10 min. The bead-bound cells were recovered with a magnet, washed four times, and the cells immediately collected for protein lysates and PIP2 ELISA protocols. All isolation steps were done in the presence of phosphatase inhibitors.

### Statistics

Statistical analysis of data was done using Microsoft Excel or Plotly. Statistical significance was set at a minimum of *p* ≤ 0.05, and is indicated in individual figure legends. Student *t*-tests, two tailed, were used when analyzing two groups within a single experiment. Box plots are graphed showing the median versus the first and third quartiles of the data (the middle, top, and bottom lines of the box, respectively) using Plotly. The whiskers demonstrate the spread of data within 1.5x above and below the interquartile range. All data points are shown as individual dots, with outliers shown above or below the whiskers.

### Study approval

Zebrafish and mouse husbandry and research protocols were reviewed and approved by the NICHD and NIDCR Animal Care and Use Committee at the National Institutes of Health. All animal studies were carried out according to NIH-approved protocols, in compliance with ethical regulations for animal testing and research outlined in the Guide for the Care and use of Laboratory Animals.

### Reporting summary

Further information on research design is available in the Nature Research Reporting Summary linked to this article.

## Supplementary information

Supplementary Information

Reporting Summary

## Data Availability

The data sets generated and/or analyzed during the current study are available from the corresponding author B.M.W. upon reasonable request. The source data underlying main and supplementary figures are provided as a Source Data file.

## Source data

Source Data
