# Supplementary material for: Anti-angiogenic effects of VEGF stimulation on endothelium deficient in phosphoinositide recycling
Source: Nat Commun. 2020 Mar 5;11:1204. doi: 10.1038/s41467-020-14956-z (PMC7058007; doi:10.1038/s41467-020-14956-z)
Supplement: Supplementary file 1 — Supplementary Information [file 41467_2020_14956_MOESM1_ESM.pdf]

Supplementary Information

Stratman, et al

Supplementary Figure 1

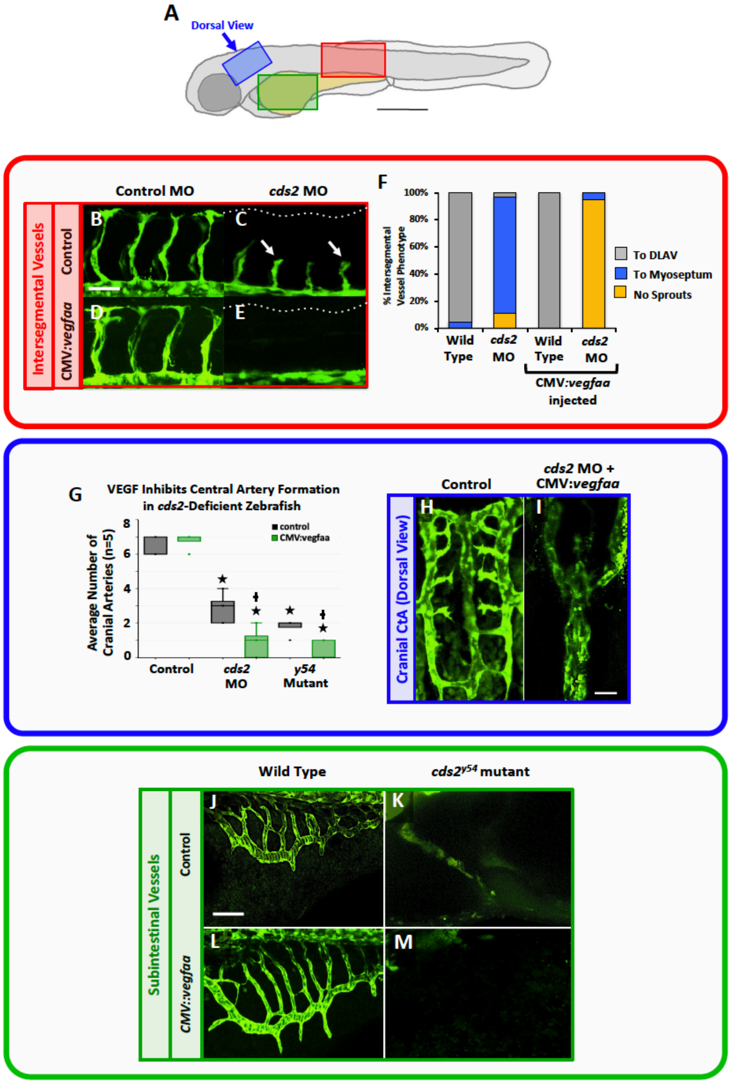

Supplementary Figure 1. *cds2* mutants and morphants challenged with exogenous *vegfaa* have enhanced angiogenic defects in multiple vascular beds. **(A)** Diagram of a zebrafish embryo[54] with the boxes highlighting the regions imaged in additional panels. The red box highlights the region imaged for B-F, the blue box highlights the region imaged for panels G-I, and green box highlights the region imaged for panels J-M. **(B-E)** Confocal images of trunk ISVs in 32 hpf *Tg(fli-EGFP)^{y1}* zebrafish control morpholino ("MO"; B,D) or *cds2* MO (C,E) injected with or control (B,C) or CMV:*vegfaa* DNA (D,E). Injection of *cds2* MO elicits a phenotype indistinguishable from *cds2* mutants, including stalling of ISV angiogenesis (white arrows in panel C), and exacerbated inhibition of angiogenesis in the presence of CMV:*vegfaa* DNA (E). **(F)** Quantitation of the ISV phenotypes of 32 hpf *Tg(fli-EGFP)^{y1}* control or *cds2* MO animals injected with control or CMV:*vegfaa* DNA. Bars show the percentage of ISV that have failed to sprout (yellow), ISV that have grown only up to the horizontal myoseptum half-way up the trunk (blue), and ISV that have grown to the dorsal trunk to form the DLAV (gray). n=100 ISV segments per condition. **(G)** Quantitation of the number of hindbrain central arteries (CtA) in 40 hpf *Tg(fli-EGFP)^{y1}* wild type control, *cds2* MO injected, or *cds2^{y54}* mutant animals that were either un-injected (black bars) or injected with CMV:*vegfaa* DNA (green bars). Overexpressing *vegfaa* similarly exacerbates CtA defects in both *cds2* morpholino-injected and *cds2^{y54}* mutant animals. n=5 animals per condition, $p \leq 0.05$; *Significance from control; +Significance from paired control. **(H,I)** Representative dorsal view confocal images of the hindbrain vasculature in 40 hpf *Tg(fli-EGFP)^{y1}* zebrafish injected with either control MO (H) or *cds2* MO + CMV:*vegfaa* DNA (I), showing loss of CtA formation in *cds2* MO + CMV:*vegfaa* DNA-injected animals. Scale bar = 100 µm. **(J-M)** Confocal images of 3 dpf *Tg(fli-EGFP)^{y1}* subintestinal vessels in wild type (J,L) or *cds2^{y54}* mutant (K,M) animals in the absence (J,K) or presence (L,M) of injected CMV:*vegfaa* DNA. Rostral is to the left and dorsal is up in all image panels except panels H,I. Panels H,I are dorsal views with rostral up and left to the left. Bars = 100 µm. Box plots are graphed showing the median versus the first and third quartiles of the data (the middle, top, and bottom lines of the box respectively). The whiskers demonstrate the spread of data within 1.5x above and below the interquartile range. All data points are shown as individual dots, with outliers shown above or below the whiskers.

Supplementary Figure 2

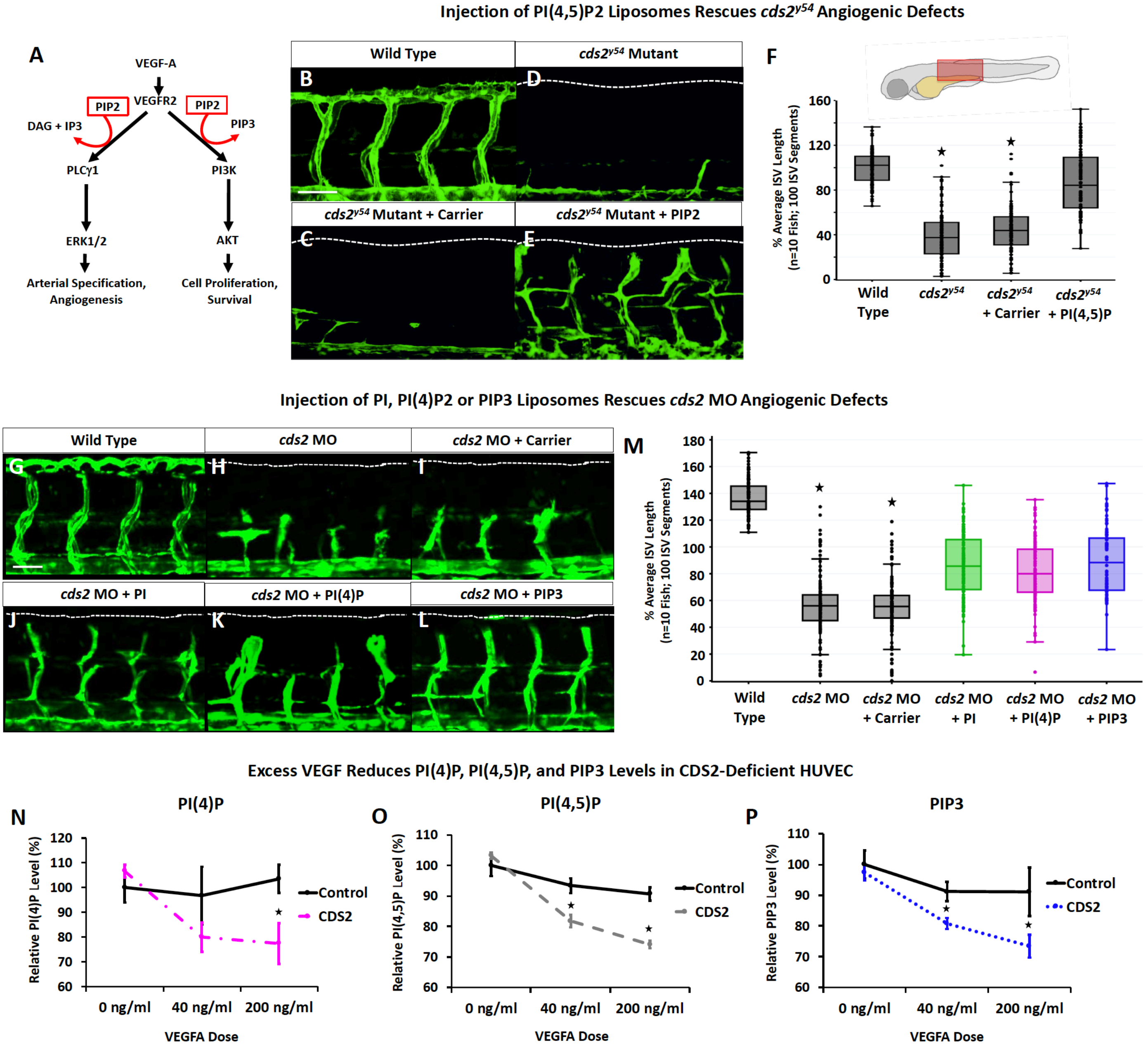

Supplementary Figure 2. PI liposome injection rescues angiogenic defects in *cds2* deficient animals. **(A)** Schematic demonstrating the requirement of PIP2 for VEGF-mediated angiogenic signaling. **(B-E)** Confocal images of ISV in 72 hpf *Tg(fli-EGFP)*^{y1} wild-type animals (B) or *cds2*^{y54} mutant siblings (C-E) that were uninjected (D) or subjected to a single intravascular injection at 48 hpf with liposome carrier (C) or liposome carrier + PIP2 (E). **(F)** Quantitation of average ISV length in *Tg(fli-EGFP)*^{y1} wild-type (column 1) or *cds2*^{y54} mutant sibling animals that were either uninjected (column 2) or subjected to a single intravascular injection at 48 hpf with liposome carrier (column 3) or liposome carrier + PIP2 (column 4). Percent average ISV length with wild type average length set to 100%. Schematic indicates the site for analysis within the zebrafish trunk. n=100 ISV segments per condition. (**G-L**) Confocal images of ISV in 72 hpf *Tg(fli-EGFP)*^{y1} wild-type animals (G) or *cds2* MO injected siblings (H-L) that were uninjected (H) or subjected to a single intravascular injection at 48 hpf with liposome carrier (I), liposome carrier + PI (J), liposome carrier + PI(4)P (K), or liposome carrier + PIP3 (L). **(M)** Quantitation of average ISV length in *Tg(fli-EGFP)*^{y1} wild-type (column 1) or *cds2* MO injected sibling animals that were either uninjected (column 2) or subjected to a single intravascular injection at 48 hpf with liposome carrier (column 3), liposome carrier + PI (column 4), liposome carrier + PI(4)P (column 5), or liposome carrier + PIP3 (column 6). (**N-P**) ELISA quantitation of PI(4)P (N), PI(4,5)P (O), or PIP3 (P) levels in control or CDS2 siRNA treated HUVECs incubated with 40, or 200 ng/ml rhVEGF-A over at a 12 hour time point, normalized to control siRNA-treated HUVEC without added VEGFA. 9 technical replicates were measured per sample, per experiment. Data is graphed as the average of 2 experimental replicates, $p \leq 0.05$, ± SD. Data is representative of two experimental replicates. *Significance from wild type (t-test). Bars = 100 µm. Box plots are graphed showing the median versus the first and third quartiles of the data (the middle, top, and bottom lines of the box respectively). The whiskers demonstrate the spread of data within 1.5x above and below the interquartile range. All data points are shown as individual dots, with outliers shown above or below the whiskers.

Supplementary Figure 3

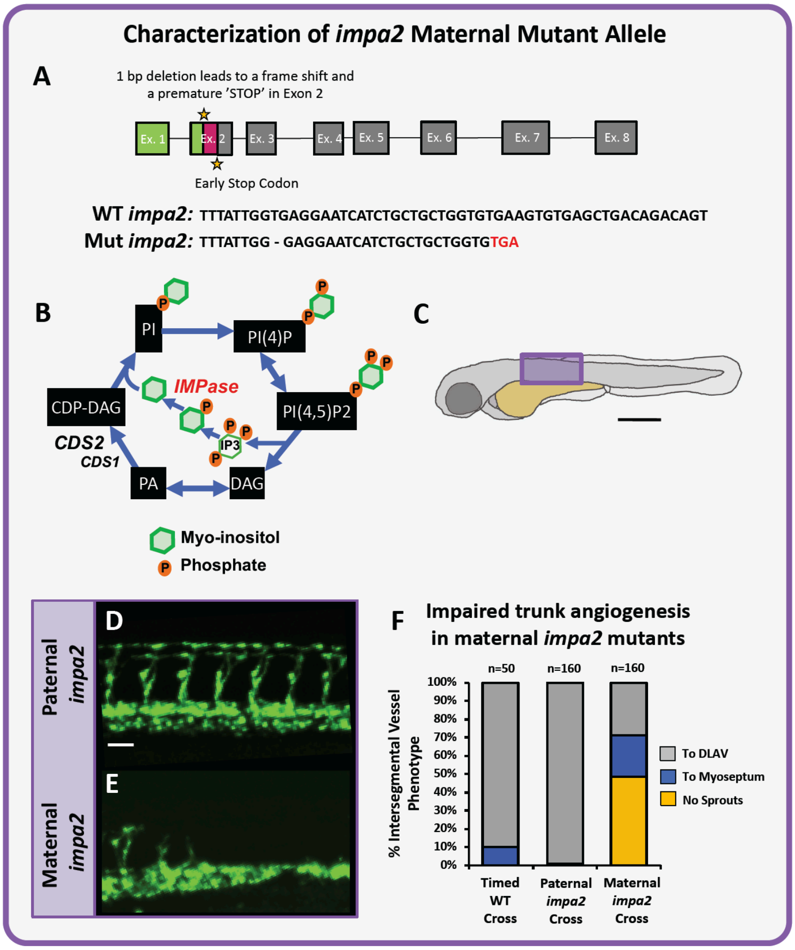

Supplementary Figure 3. Phenotype and allelic characterization of the *impa2*^{y602} zebrafish mutant. (A) Gene structure and sequence of the *impa2*^{y602} mutant generated for this study using CRISPR/Cas9 mutagenesis. A 1 base pair deletion in exon 2 at 144bp leads to a frame shift and early termination after 169bp. **(B)** Schematic diagram of phosphoinositide recycling. CDS1, CDS2, and IMPase enzymes facilitate regeneration of phosphoinositol after consumption of PIP2 (see **Fig. S13** for details). **(C)** Diagram of a zebrafish embryo[54] with the purple box highlighting the region imaged in panels D-F. **(D-F)** Zebrafish *impa2*^{y602} mutants were generated by injection of CRISPR/cas9 target to exon 3 of the *impa2* gene, followed by subsequent identification of the described allele (A). Viable homozygous *impa2*$^{y602/y602}$ animals can be generated by crossing together two *impa2*$^{y602/+}$ heterozygotes, allowing for the generation of progeny depleted of their maternal *impa2* contribution. **(D,E)** Confocal images of trunk ISVs in 32 hpf *Tg(fli-EGFP)*^{y1} zebrafish from crosses between *impa2*^{y602} homozygous males and heterozygous females (D) or between *impa2*^{y602} homozygous females and heterozygous males (E), showing that lack of maternally contributed *impa2* leads to trunk angiogenesis defects. **(F)** Quantitation of the ISV phenotypes in 32 hpf *Tg(fli-EGFP)*^{y1} zebrafish from (D) and (E), versus a timed controlled clutch of embryos. Bars show the percentage of ISV that have failed to sprout (yellow), ISV that have grown only up to the horizontal myoseptum half-way up the trunk (blue), and ISV that have grown to the dorsal trunk to form the DLAV (gray). n= number of ISV sprouts included in analysis (shown above the bars for each condition). Data is representative of two independent experiments. Bars = 100 µm.

Supplementary Figure 4

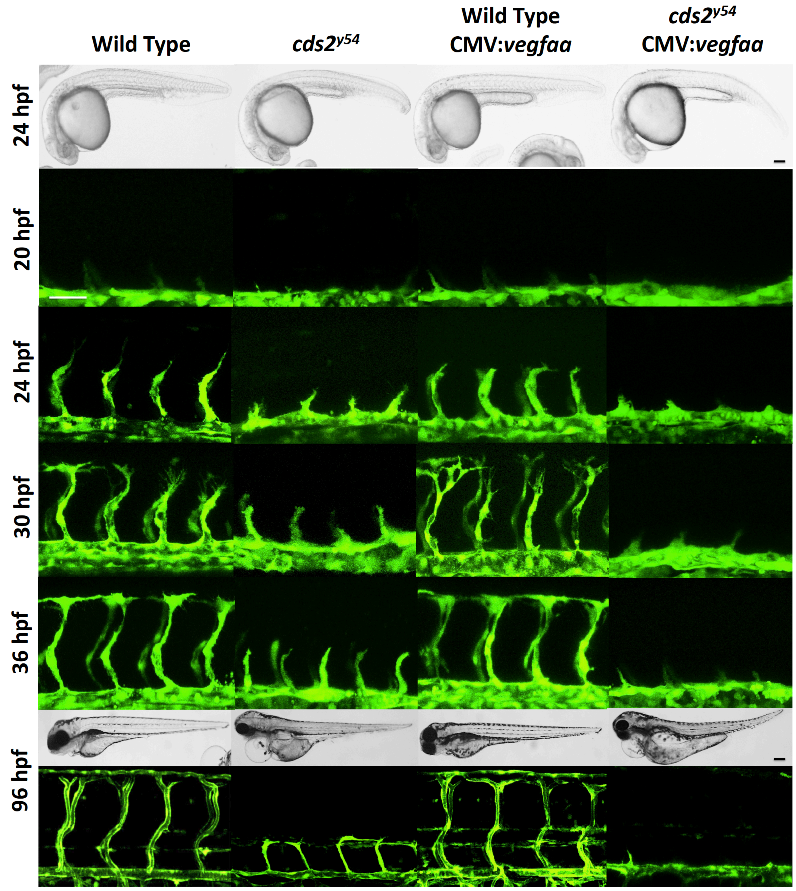

Supplementary Figure 4. Exogenous *vegfaa* stimulation causes stalling of angiogenic sprouts. Confocal images of trunk vascular phenotypes in control and CMV:*vegfaa* injected wild type or *cds2^{y54}* mutants at 20, 24, 30, 36 and 96 hpf. Column I: control DNA, wild type, Column II: control DNA, *cds2^{y54}* mutant, Column III: CMV:*vegfaa* DNA, wild type, Column IV: CMV:*vegfaa* DNA, *cds2^{y54}* mutant. Representative images from 10 embryos per condition, and two experimental replicates. Bars = 100 µm.

Supplementary Figure 5

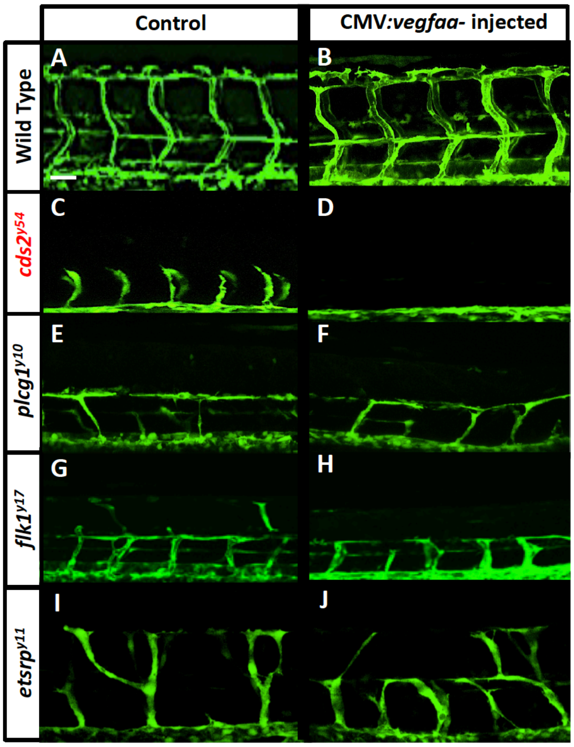

Supplementary Figure 5. Exogenous *vegfaa* stimulation uniquely exacerbates vascular defects in *cds2*^{y54} mutants. (A-J) Confocal images of trunk vascular phenotypes in control (A,C,E,G,I) and CMV:*vegfaa* injected (B,D,F,H,J) 36 hpf *Tg(fli-EGFP)*^{y1} transgenic zebrafish embryos that were either wild type (A,B), *cds2*^{y54} mutant (C,D), *plcγ1*^{y10} mutant (E,F), *flk1*^{y17} mutant (G,H), or *etsrp*^{y11} mutant (I,J). Only *cds2*^{y54} mutants show exacerbated vascular defects following injection of CMV:*vegfaa*. Representative images from 10 embryos per condition, and 3 experimental replicates. Bars = 100 µm.

Supplementary Figure 6

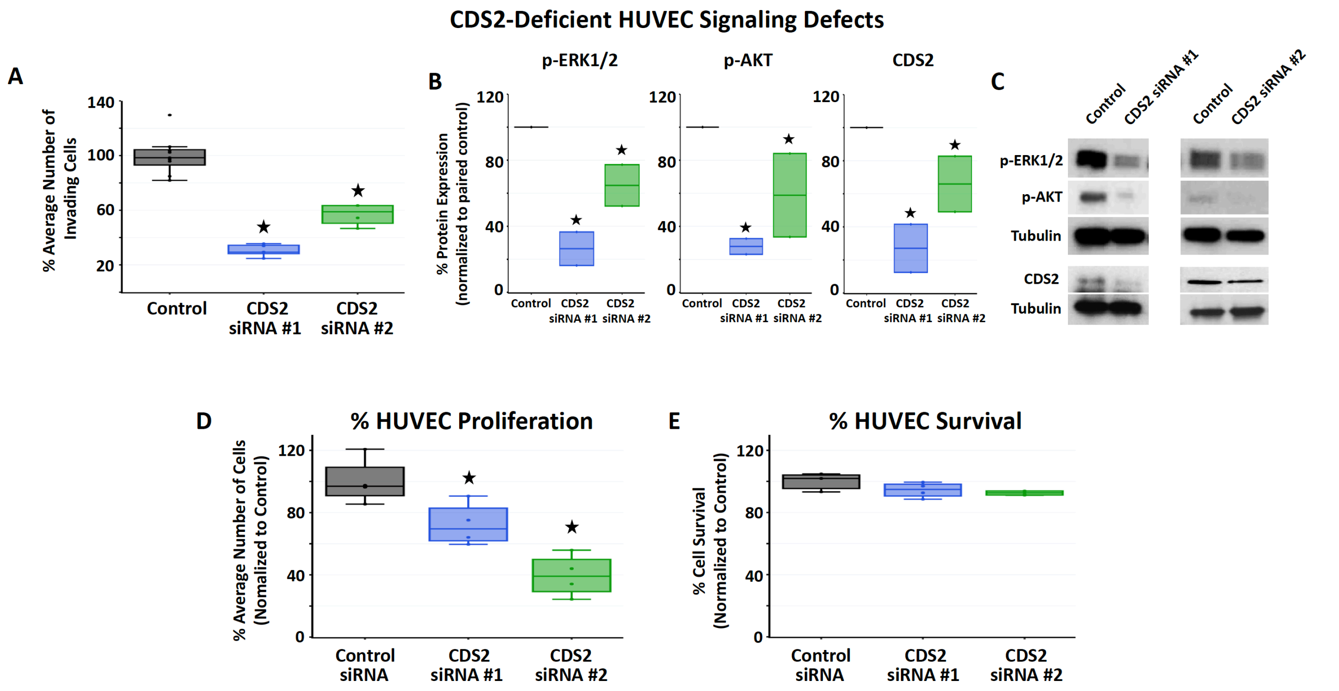

Supplementary Figure 6. *In vitro* defects of CDS2 deficient endothelium and CDS2 siRNA validation. **(A)** Two single siRNA targets of CDS2 were chosen for analysis. HUVECs treated with control or the CDS2 siRNAs were plated on top of collagen type I gel and cellular invasion into the collagen gel quantified at 18 hours. **(B,C)** Western blot analysis of HUVECs treated with control or the individual CDS2 siRNA targets. Lysates were collected following incubation with VEGFA and p-ERK1/2, p-AKT and CDS2 protein levels analyzed. Quantification (B) and representative blots (C) for each of the siRNAs are shown. Analysis of CDS2 protein levels shows approximately 75-80% protein suppression using CDS2 siRNA target #1, so it was chosen for use in all of the main text figures. **(D,E)** Proliferation (D) and apoptosis (Total cell number – Caspase 3 positive cells; E) were analyzed in cells treated with control or the individual CDS2 siRNA targets. All data is presented as a percentage of the control siRNA treated cells, and is representative of 3 experimental replicates. $p \leq 0.05$, error bars ± SD; *Significance from control (t-test). Box plots are graphed showing the median versus the first and third quartiles of the data (the middle, top, and bottom lines of the box respectively). The whiskers demonstrate the spread of data within 1.5x above and below the interquartile range. All data points are shown as individual dots, with outliers shown above or below the whiskers.

Supplementary Figure 7

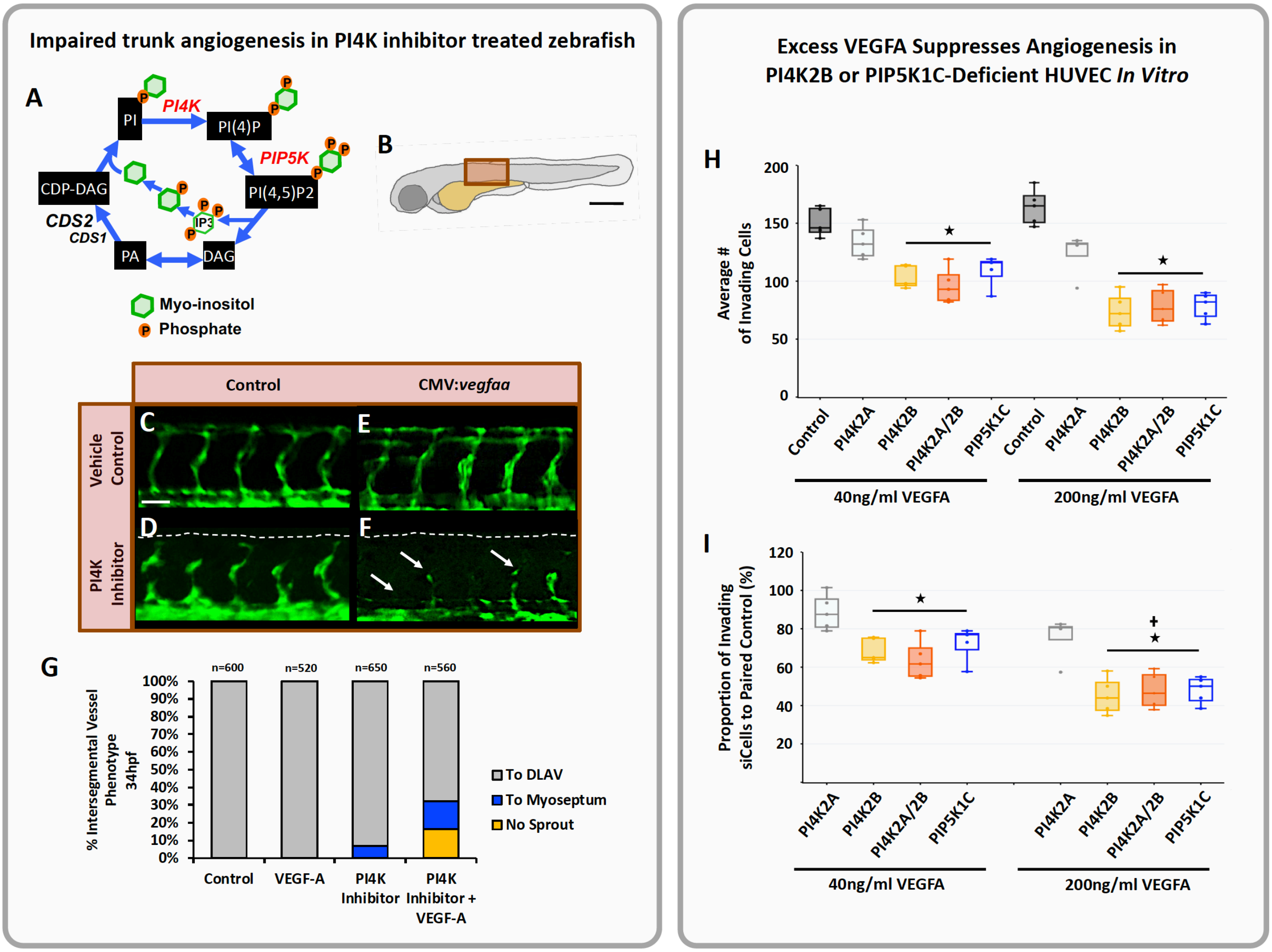

Supplementary Figure 7. *In vitro* and *in vivo* defects of PI4K and PIP5K1C deficient endothelium. **(A)** Schematic diagram of phosphoinositide recycling. PI4K and PIP5K kinases phosphorylate PI and the 4 and 5 positions respectively to generate PI(4,5)P2 (see **Fig. S12** for details). **(B)** Diagram of a zebrafish embryo[54] with the red box highlighting the region imaged in panels C-F. **(C-F)** Confocal images of trunk ISVs in 34 hpf *Tg(fli-EGFP)*^{y1} zebrafish vehical control (C,E) or PI4K inhibitor (D,F) treated embryos without (C,D) or with (E,F) injected CMV:*vegfaa* DNA. Addition of CMV:*vegfaa* DNA to zebrafish treated with PI4K inhibitor causes stalling of angiogenesis (white arrows in panel F). **(G)** Quantitation of the ISV phenotypes in 34 hpf *Tg(fli-EGFP)*^{y1} vehicle control or PI4K inhibitor treated animals with or without injected CMV:*vegfaa* DNA. Bars show the percentage of ISV that have failed to sprout (yellow), ISV that have grown only up to the horizontal myoseptum half-way up the trunk (blue), and ISV that have grown to the dorsal trunk to form the DLAV (gray). n= number of ISV sprouts included in analysis (shown above the bars for each condition). Data is representative of two independent experiments. **(H,I)** Quantification of HUVEC cellular invasion into collagen gels at VEGFA doses indicated. HUVEC were treated with control siRNA or siRNA targeting PI4K2A, PI4K2B, PI4K2A+ PI4K2B, or PIP5K1C. Graphs of the same data are plotted both as the average number of invading cells compared to control siRNA-treated HUVEC (H), and as the relative percentage of cellular invasion normalized to VEGFA dose-matched controls (see methods; I). n=4 collagen gels; data is representative of 2 independent experiments. *Significance from control; +Significance from 40ng/ml VEGFA dose (t-test). Bars = 100 µm. Box plots are graphed showing the median versus the first and third quartiles of the data (the middle, top, and bottom lines of the box respectively). The whiskers demonstrate the spread of data within 1.5x above and below the interquartile range. All data points are shown as individual dots, with outliers shown above or below the whiskers.

Supplementary Figure 8

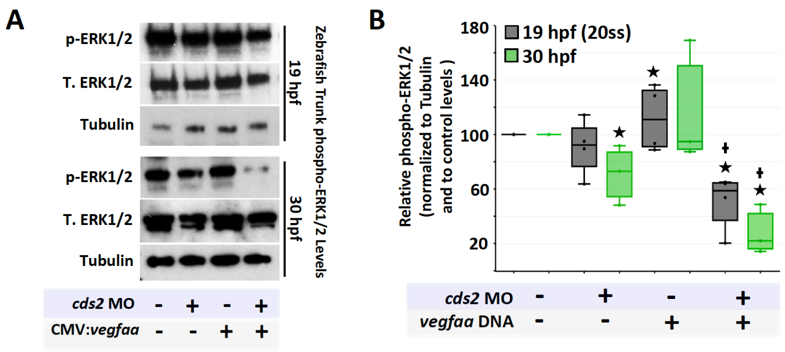

Supplementary Figure 8. VEGFA inhibits ERK1/2 signaling in CDS2 deficient endothelium. (A) Representative western blots and **(B)** quantitation of ERK1/2 phosphorylation in zebrafish trunk tissue collected at 19 hpf (black bars) or 30 hpf (green bars) from animals injected with (i) control MO, (ii) *cds2* MO, (iii) CMV:*vegfaa* DNA, or (iv) *cds2* MO + CMV:*vegfaa* DNA. Bars indicate the ratio of pERK1/2 to tubulin levels for each condition, normalized to the phospho-ERK1/2/tubulin levels of the control MO injected animals (set at 100%). Data is averaged from 3 experimental replicates. $p \leq 0.05$, error bars ± SEM; *Significance from control; +Significance from *cds2* MO alone condition (t-test). Box plots are graphed showing the median versus the first and third quartiles of the data (the middle, top, and bottom lines of the box respectively). The whiskers demonstrate the spread of data within 1.5x above and below the interquartile range. All data points are shown as individual dots, with outliers shown above or below the whiskers.

Supplementary Figure 9

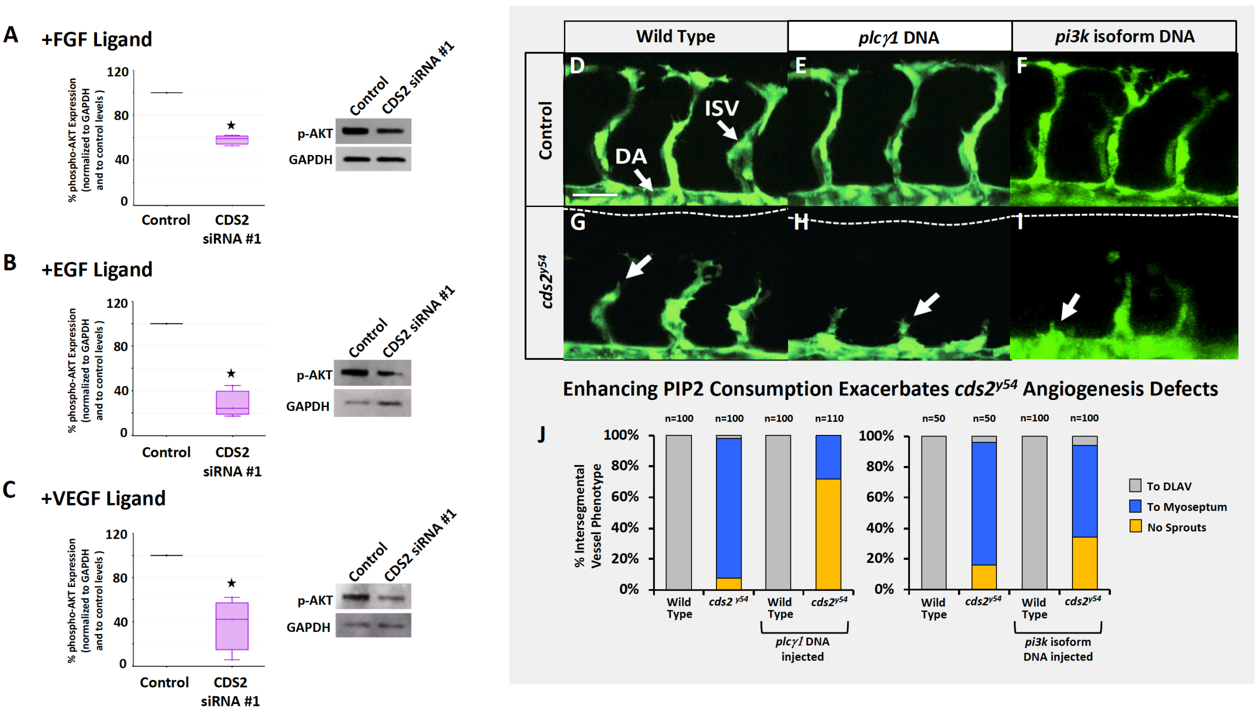

Supplementary Figure 9. Pro-angiogenic signaling becomes anti-angiogenic in the presence of compromised PI recycling. (A-C) Western blot analysis of HUVECs treated with control or CDS2 siRNA #1 target. Protein lysates were collected following 10 min incubation with FGF, EGF, or VEGFA and p-AKT activation levels analyzed. Quantification (left) and representative blots (right) for each of the treatment conditions are shown compared to GAPDH loading control. All data is represented as a percentage of the control siRNA treated cells. n=3 biologically independent experimental replicates per condition. $p \leq 0.05$, error bars ± SEM; *Significance from control (t-test). Box plots are graphed showing the median versus the first and third quartiles of the data (the middle, top, and bottom lines of the box respectively). The whiskers demonstrate the spread of data within 1.5x above and below the interquartile range. All data points are shown as individual dots, with outliers shown above or below the whiskers. **(D-I)** Confocal images of mid-trunk ISV in 32 hpf *Tg(fli-EGFP)^{y1}* wild-type (D-F) or *cds2^{y54}* mutant (G-I) animals injected with control (D,G), *plcg1* (E,H), or mixed *p110δ* and *pi3k-c2β* (*pi3k* isoform; F,I) DNA. Arrows in panel D point to dorsal aorta (DA) and intersegmental vessel sprouts (ISV); white arrows in panels G-I indicate stalled or absent ISV sprouts. **(J)** Quantitation of the ISV phenotypes of 32 hpf *Tg(fli-EGFP)^{y1}* wild-type or *cds2* MO morphant animals injected with control, *plcg1,* or *pi3k* isoform DNA. The bars show the percentages of ISV that have failed to sprout (yellow), ISV that have grown only up to the horizontal myoseptum half-way up the trunk (blue), and ISV that have grown all the way to the dorsal trunk to form the DLAV (gray). The number of segments counted is shown above each bar on the graphs. Data is representative of 3 experimental replicates. Bars = 100 µm.

Supplementary Figure 10

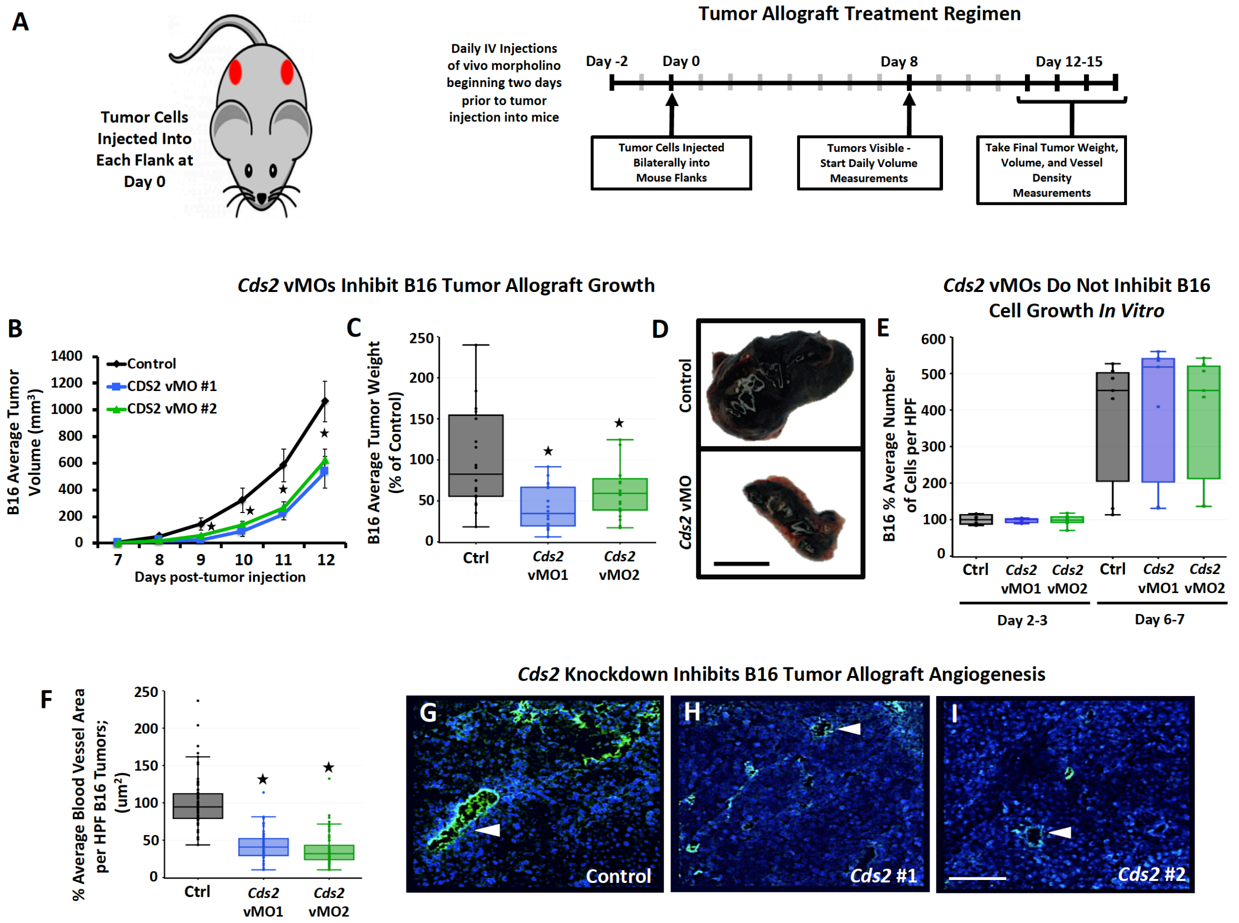

Supplementary Figure 10. Systemic *Cds2* suppression inhibits tumor growth in B16-F10 allograft models. (A) Schematic of tumor allograft assay. B6/C57 adult mice received daily intravenous injections of control vivoMorpholino (vMO) or vMO targeting one of two independent sites in the *Cds2* gene, starting two days prior to tumor implantation. B16-F10 melanoma tumor cells were injected into each flank of the mice at day 0 and tumors allowed to develop for 12-15 days. Volume measurements were taken daily when tumors became visible with final tumor volumes and weights, mouse weight, and tumor blood vessel density measurements taken at the termination of the experiment. **(B-D)** B16-F10 melanoma tumor volume (B), final tumor weight (C), representative B16-F10 tumors from control versus vMO treated animals (D). Quantitation of average tumor volume (in mm^3) of B16-F10 tumors, averaging the measurements of 10 tumors per treatment group. Quantitation of average tumor weight (in mg) of B16-F10 tumors at 12 days post-tumor implantation, averaging the measurements of 10 biologically independent tumors per treatment group. $p \leq 0.05$, error bars ± SEM. *Significance from control (t-test); Bar = 1 cm in panel D. **(E)** B16-F10 melanoma cell proliferation after 2 or 7 days of growth *in vitro*. Quantitation of the average number of B16-F10 tumor cells per high power field, measured 2 or 7 days (approximately 3 and 14 division cycles) after seeding 5,000 cells per well, with vMO doses comparable to those administered to the mice. Data is representative of 3 independent experimental replicates. **(F)** Quantitation of B16-F10 tumor vessel density (F) in *Cds2* vMO- versus control vMO-treated mice. For all vascular density measurement experiments three images per tumor were acquired (see tumor numbers above) and vascular density measured for all groups. A minimum of 2 slide sections from each tumor (taken from sections at least 10 slices apart) were utilized for analysis. **(G-I)** Representative images of CD31/PECAM labeled (green) B16-F10 tumor sections from control vMO (G) and *Cds2* vMO (H,I) treated mice. White arrowheads indicate sites of CD31/PECAM positive blood vessel labeling. Bar = 100 µm. $p \leq 0.05$, error bars ± SEM. *Significance from control (t-test). Box plots are graphed showing the median versus the first and third quartiles of the data (the middle, top, and bottom lines of the box respectively). The whiskers demonstrate the spread of data within 1.5x above and below the interquartile range. All data points are shown as individual dots, with outliers shown above or below the whiskers.

Supplementary Figure 11

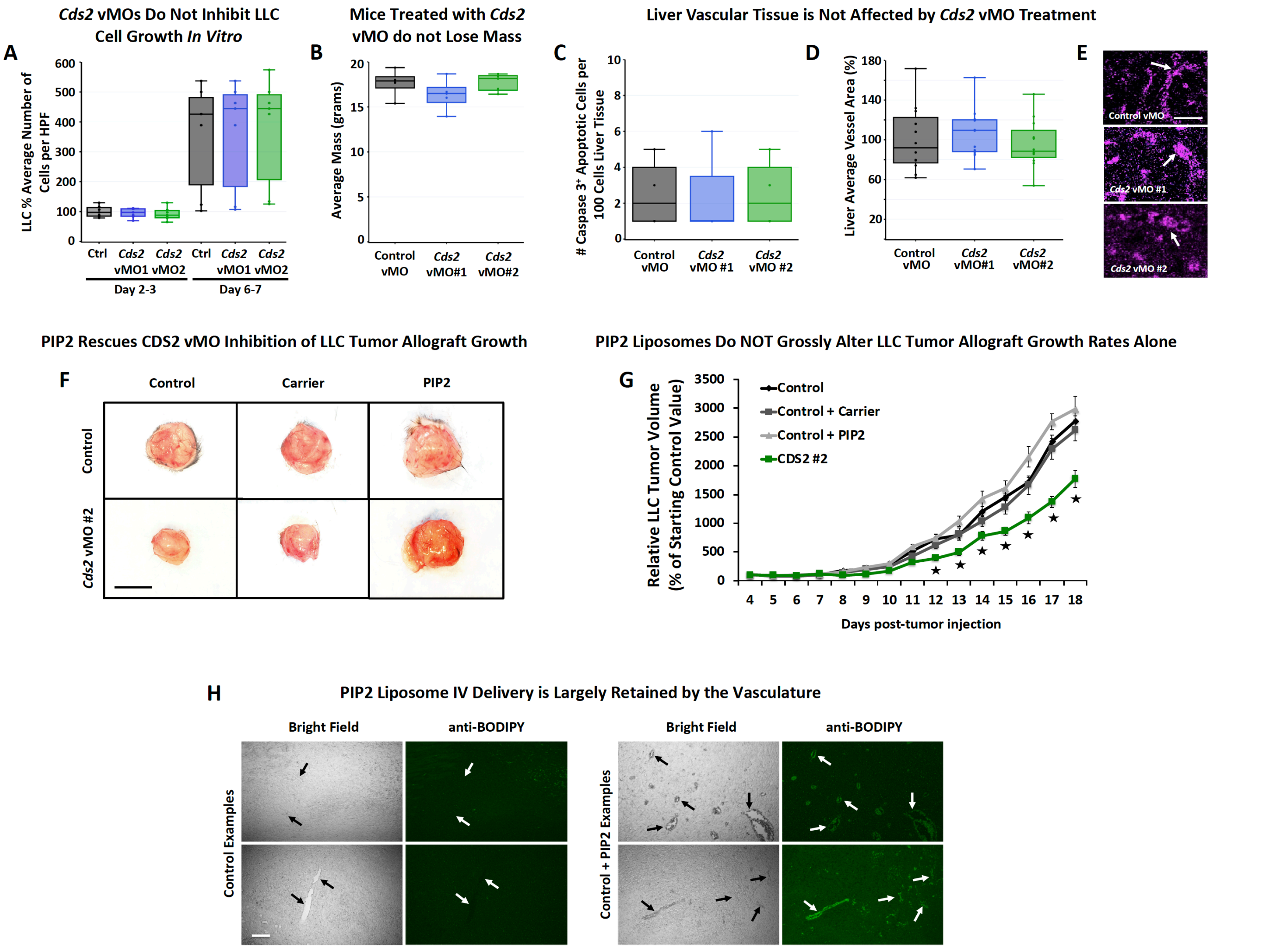

Supplementary Figure 11. Systemic *Cds2* suppression inhibits tumor growth in LLC allograft tumor models but does not affect normal tissue. (A) Quantitation of the average number LLC tumor cells per high power field, measured 2 or 7 days (approximately 3 and 14 division cycles) after seeding 5,000 cells per well, with vMO doses comparable to those administered to the mice. Data is representative of 3 independent experimental replicates. **(B)** Quantitation of final mass (in grams) of mice treated with either control or *Cds2*-specific vMOs. No significant decrease in mass was noted following treatment with *Cds2* vMOs. n=5 mice per group. **(C)** Average number of caspase 3+ cells per 100 cells in liver tissue collected from control versus *Cds2* vMO treated animals. No significant change was noted. n=4 biologically independent livers, 3 images per liver section, and 2 sections per liver were analyzed for each treatment group. **(D,E)** Analysis of vessel density in liver tissue collected from control versus *Cds2* vMO treated animals. (D) Quantification and (E) representative images of CD31/PECAM labeled vessels (purple, highlighted by white arrows) are shown for comparison. Data is represented as a percentage of control. No significant changes were noted. For all vascular density measurement experiments: three images per liver were acquired (see liver numbers above) and vascular density measured for all groups. A minimum of 2 slide sections from each liver (taken from sections at least 10 slices apart) were utilized for analysis. Bar = 100 μm. **(F)** Representative images of tumors collected from control vMO treated animals or *Cds2* vMO treated animals co-administered nothing, carrier or PIP2 liposomes at 18 days post-tumor implantation. Bar =1 cm. **(G)** Average percent volume measurements (in mm^3) of control (n=8 biologically independent tumors), control + carrier (n=9 biologically independent tumors), and control + PIP2 (n=8 biologically independent tumors) versus *Cds2* vMO #2 (n=9 biologically independent tumors) treatment conditions. $p \le 0.05$, error bars ± SEM. *Significance from control (t-test). **(H)** To confirm effective delivery of PIP2 liposomes to the tumor vasculature, BODIPY-labeled PIP2 liposomes were injected into the mice as part of our studies. Anti-BODIPY immunostaining of fixed tumor sections reveals enrichment of PIP2 levels in the vasculature of injected animals (right panels, GFP) versus control uninjected animals (left panels, GFP), and compared to the surrounding tumor tissue. Bright field panels are included to demonstrate where the blood vessels are located within the tumor (arrows). Two examples for the control and the PIP2 injected condition are shown as representative examples. Bars = 100 μm. Box plots are graphed showing the median versus the first and third quartiles of the data (the middle, top, and bottom lines of the box respectively). The whiskers demonstrate the spread of data within 1.5x above and below the interquartile range. All data points are shown as individual dots, with outliers shown above or below the whiskers.

Supplementary Figure 12

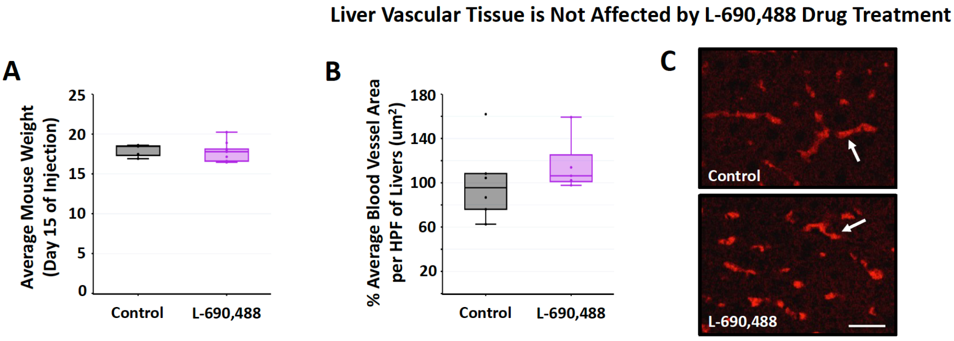

Supplementary Figure 12. L-690,488 treatment does not affect normal tissue. (A) Quantitation of final mass (in grams) of mice treated with either control (DMSO, n=5) or L-690,488 (n=10). No significant decrease in mass was noted following treatment with the chemical inhibitor. **(B,C)** Analysis of vessel density in liver tissue collected from control (DMSO, n=4 biologically independent livers) versus L-690,488 (n=5 biologically independent livers) treated animals. (B) Quantification and (C) representative images of CD31/PECAM labeled vessels (red, highlighted by white arrows) are shown for comparison. Data is represented as a percentage of control. Bars = 100 µm. Box plots are graphed showing the median versus the first and third quartiles of the data (the middle, top, and bottom lines of the box respectively). The whiskers demonstrate the spread of data within 1.5x above and below the interquartile range. All data points are shown as individual dots, with outliers shown above or below the whiskers. No significant changes were noted.

Supplementary Figure 13

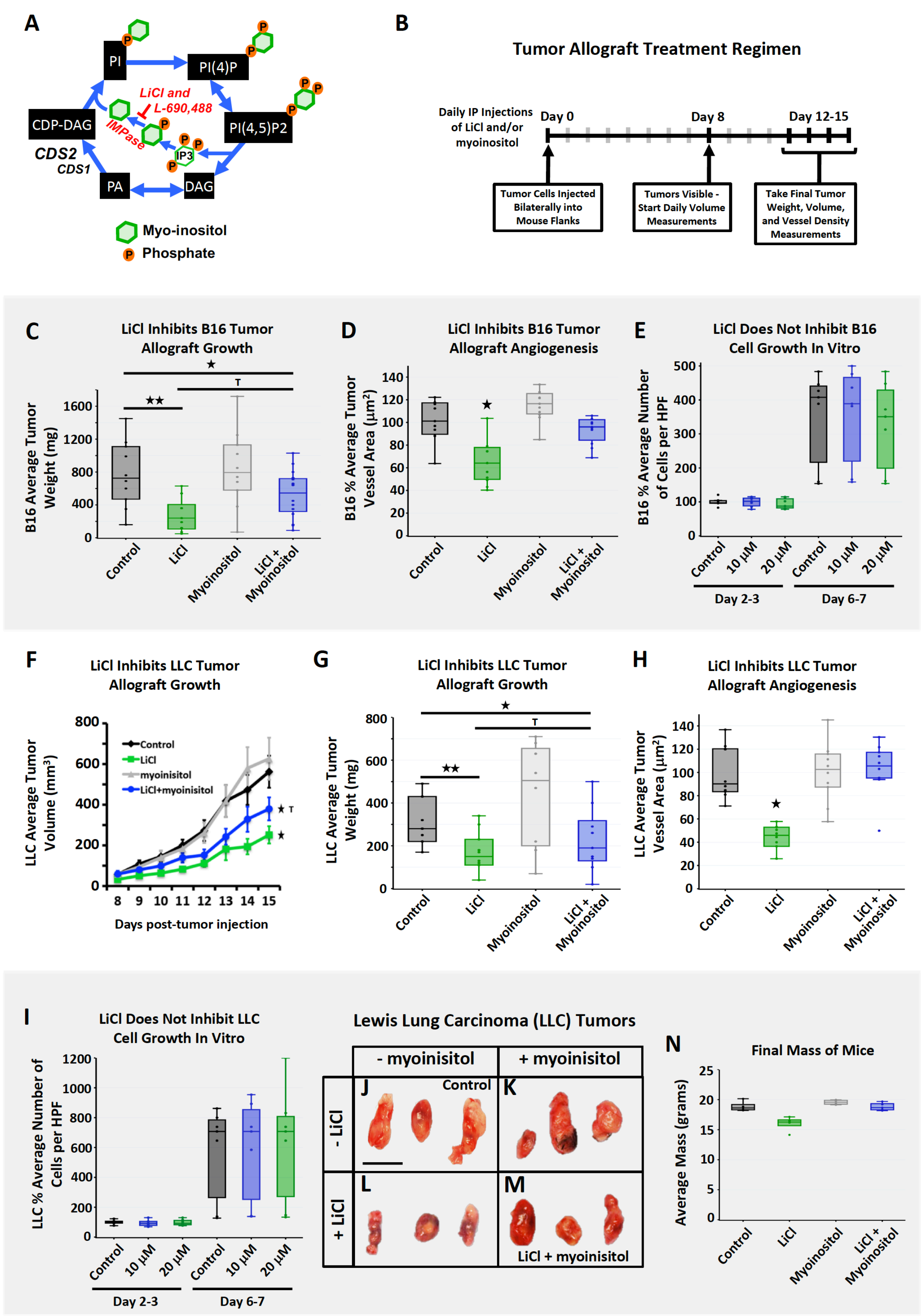

Supplementary Figure 13. Lithium chloride inhibits B16-F10 and LLC tumor growth and tumor vascularization. (A) Schematic diagram of the blocking effect of LiCl on inositol-monophosphatase (IMPase), an enzyme necessary for recycling the myoinositol rings required to regenerate PI and its isoforms. Addition of myoinositol, the product of the reaction catalyzed by IMPase, can be used to "rescue" the effects of IMPase suppression, revealing IMPase-specific effects of LiCl as opposed to other, off-target effects (that are not rescued by myoinositol). **(B)** Schematic of tumor allograft assay for LiCl treatment experiments. B16-F10 melanoma or Lewis Lung Carcinoma (LLC) tumor cells were injected into each flank of the mice at day 0 and tumors were allowed to develop for 12-15 days with daily IP injections of LiCl and/or myoinositol. Tumor volume measurements were taken daily starting at day 8, when tumors became visible, with final tumor volumes and weights,

mouse weight, and tumor blood vessel density measurements taken at the termination of the experiment. **(C-E)** B16-F10 melanoma final tumor weight (C, n=10 biologically independent tumors), tumor vessel density (D, n=10 biologically independent tumors), and number of B16-F10 melanoma cells present after 2 or 7 days of growth *in vitro* (E). **(F-I)** LLC tumor volume (F, n=10 biologically independent tumors), final tumor weight (G, n=8 biologically independent tumors), tumor vessel density (H), and number of LLC cells present after 2 or 7 days of growth *in vitro* (I). **(F)** Quantitation of average LLC tumor volume (in mm^3), averaging the measurements of all tumors per treatment group. **(C,G)** Quantitation of average tumor weight (in mg) of B16-F10 (C) or LLC (G) tumors at 12 days post-tumor implantation, averaging the measurements of all tumors per treatment group. **(D,H)** Quantitation of B16-F10 (D) or LLC (H) tumor vessel density in control mice vs. LiCl treated animals (For all vascular density measurement experiments: three images per tumor were acquired (see tumor numbers above) and vascular density measured for all groups. A minimum of 2 slide sections from each tumor (taken from sections at least 10 slices apart) were utilized for analysis.). **(E,I)** Quantitation of the average number of B16-F10 (E) or LLC (I) tumor cells per high power field, measured 2 or 7 days (approximately 3 and 14 division cycles) after seeding 5,000 cells per well, with a LiCl dose estimated to be comparable to that administered to the mice (10µM). Data is representative of 3 independent experimental replicates. **(J-M)** Representative images of LLC tumors from control (J), myoinositol treated (K), LiCl treated (L), or LiCl + myoinositol treated (M) mice. Bars = 1 cm. **(N)** Final total mass of mice from panel G, n=5. $p \leq 0.05$, error bars ±SEM; *Significance from control; ^{T} Significance from LiCl treatment condition (t-test). Box plots are graphed showing the median versus the first and third quartiles of the data (the middle, top, and bottom lines of the box respectively). The whiskers demonstrate the spread of data within 1.5x above and below the interquartile range. All data points are shown as individual dots, with outliers shown above or below the whiskers. **Abbreviations:** inositol-monophosphatase (IMPase), Diacylglycerol (DAG), Phosphatidylinositol (PI), Phosphatidic acid (PA), inositol triphosphate (IP3), phosphatidylinositol-4,5-bisphosphate (PIP2), CDP-diacylglycerol synthetase (CDS).

Supplementary Figure 14

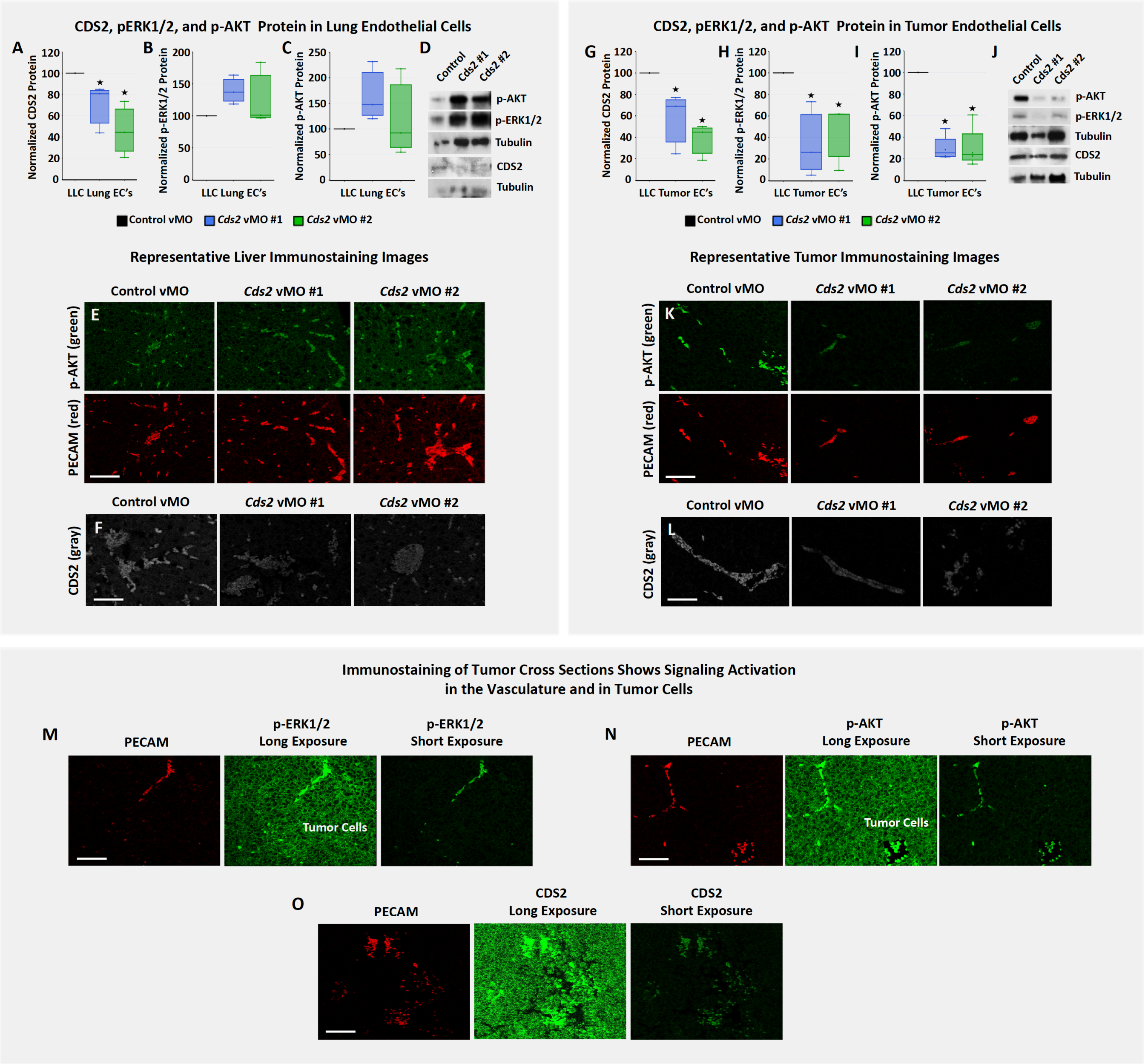

Supplementary Figure 14. Systemic *Cds2* suppression inhibits VEGFR2 downstream signaling in LLC tumor models. (A-D) Quantification of CDS2 (A), pERK1/2 (B), and pAKT (C) protein levels in ECs isolated from the lungs of LLC allografted animals. Data is generated from three western blots and normalized to tubulin levels. $p \leq 0.05$, error bars ±SEM. *Significance from control (t-test). (D) Representative western blot images from lung isolated ECs. CDS2 western blot for lung ECs is shown from a separate run of the same samples and is presented with its respective tubulin loading control. **(E,F)** Representative images of liver cross sections from the same control vMO or CDS2 vMO treated mice, immunostained with pAKT antibody (E, green images) or CDS2 antibody (F, gray images). **(G-J)** Quantification of CDS2 (G), pERK1/2 (H), and pAKT (I) protein levels in ECs isolated from LLC allografted tumors. Data is generated from three western blots and normalized to total tubulin levels. $p \leq 0.05$, error bars ±SEM. *Significance from control (t-test). (J) Representative western blot images from tumor isolated ECs. CDS2 western blot for tumor ECs is shown from a separate run of the same samples and is presented with its respective tubulin loading control. **(K,L)** Representative images of LLC tumor cross sections from the same control vMO or *Cds2* vMO treated mice, immunostained with pAKT antibody (K, green images) or CDS2 antibody (L, gray images). (**M-O**) Representative images of sections from LLC control tumors co-immunostained with CD31/PECAM (red) together with either p-ERK1/2 (M), pAKT (N), or CDS2 (O) antibody (green images). Paired longer and shorter exposure images are shown to illustrate that p-ERK1/2, p-AKT, and CDS2 staining is present in both the tumor-associated vasculature and in the tumor cells themselves, albeit to a much greater extent in the tumor vessels. For all immunostaining quantitation experiments: three images per tumor or liver were acquired and signal intensity measured for all groups. A minimum of 2 slide sections from each tumor or liver (taken from sections at least 10 slices apart) were utilized for analysis. Tumors, n=10; Livers, n=5. Bars = 200 µm. Box plots are graphed showing the median versus the first and third quartiles of the data (the middle, top, and bottom lines of the box respectively). The whiskers demonstrate the spread of data within 1.5x above and below the interquartile range. All data points are shown as individual dots, with outliers shown above or below the whiskers.

Supplementary Figure 15

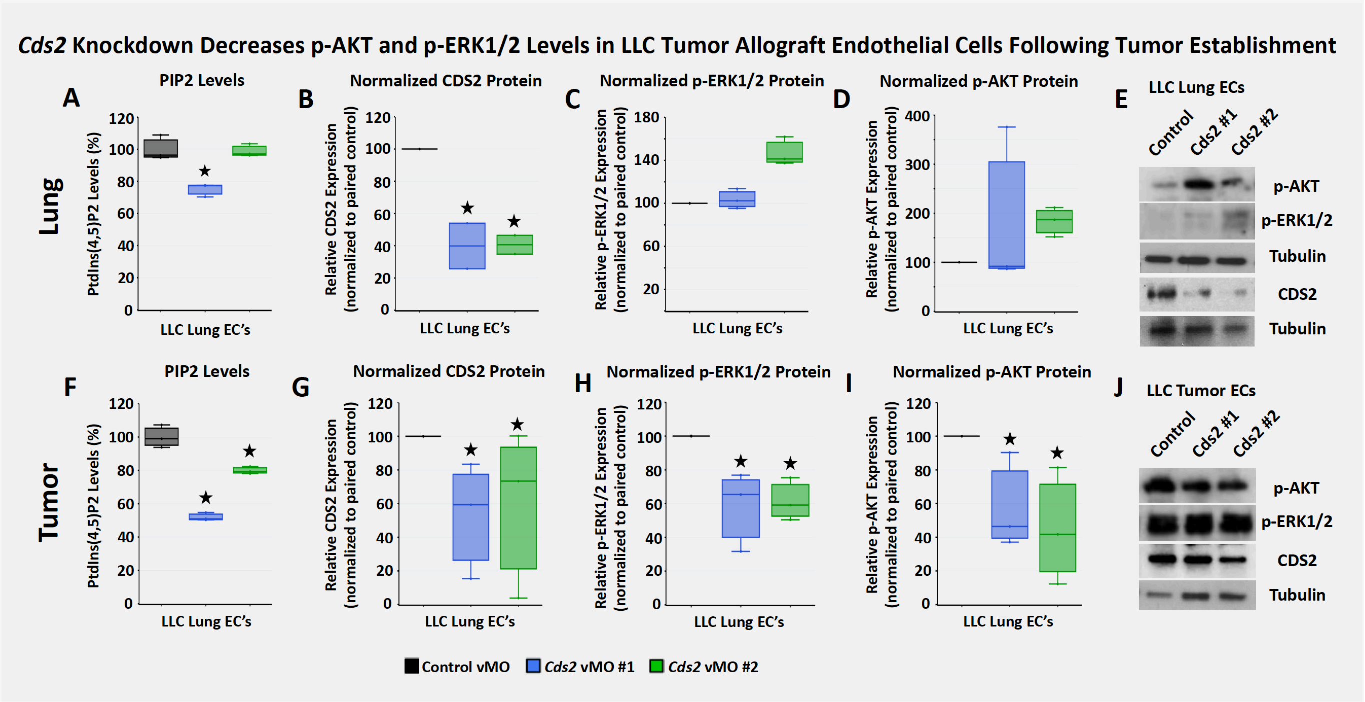

Supplementary Figure 15. Systemic CDS2 suppression inhibits VEGFR2 downstream signaling in LLC established tumors. **(A,F)** ELISA measurement of PIP2 levels in endothelial cells isolated from lungs (A) or tumors (F) from the same LLC allografted animals. Data per treatment condition is graphed as the average of 3 replicates. The ELISA measurements are normalized to whole endothelial cell tubulin lysates collected at the start of the lipid isolation procedure. **(B-E, G-J)** Quantification of CDS2 (B,G), pERK1/2 (C,H), and pAKT (D,I) protein levels and representative western blots (E,J) on ECs isolated from the lungs (B-E) or tumors (G-J) of the same LLC allografted animals. Data is generated from three western blots, except panel B which only has two experimental replicates, and normalized to tubulin levels. $p \leq 0.05$, error bars ±SEM. *Significance from control (t-test). Box plots are graphed showing the median versus the first and third quartiles of the data (the middle, top, and bottom lines of the box respectively). The whiskers demonstrate the spread of data within 1.5x above and below the interquartile range. All data points are shown as individual dots, with outliers shown above or below the whisker.
