# Supplementary material for: Anti-angiogenic effects of VEGF stimulation on endothelium deficient in phosphoinositide recycling
Source: Nat Commun. 2020 Mar 5;11:1204. doi: 10.1038/s41467-020-14956-z (PMC7058007; doi:10.1038/s41467-020-14956-z)
Supplement: Supplementary file 2 — Reporting Summary [file 41467_2020_14956_MOESM2_ESM.pdf]

natureresearch

Corresponding author(s): *Double-blind peer review submissions: write DBPR and your manuscript number here instead of author names.*

Reporting Summary

Nature Research wishes to improve the reproducibility of the work that we publish. This form provides structure for consistency and transparency in reporting. For further information on Nature Research policies, see Authors & Referees and the Editorial Policy Checklist.

Statistical parameters

When statistical analyses are reported, confirm that the following items are present in the relevant location (e.g. figure legend, table legend, main text, or Methods section).

n/a	Confirmed	
☐	☒	The exact sample size (*n*) for each experimental group/condition, given as a discrete number and unit of measurement
☐	☒	An indication of whether measurements were taken from distinct samples or whether the same sample was measured repeatedly
☐	☒	The statistical test(s) used AND whether they are one- or two-sided<br>*Only common tests should be described solely by name; describe more complex techniques in the Methods section.*
☒	☐	A description of all covariates tested
☒	☐	A description of any assumptions or corrections, such as tests of normality and adjustment for multiple comparisons
☐	☒	A full description of the statistics including central tendency (e.g. means) or other basic estimates (e.g. regression coefficient) AND variation (e.g. standard deviation) or associated estimates of uncertainty (e.g. confidence intervals)
☐	☒	For null hypothesis testing, the test statistic (e.g. *F*, *t*, *r*) with confidence intervals, effect sizes, degrees of freedom and *P* value noted<br>*Give P values as exact values whenever suitable.*
☒	☐	For Bayesian analysis, information on the choice of priors and Markov chain Monte Carlo settings
☒	☐	For hierarchical and complex designs, identification of the appropriate level for tests and full reporting of outcomes
☒	☐	Estimates of effect sizes (e.g. Cohen's *d*, Pearson's *r*), indicating how they were calculated
☐	☒	Clearly defined error bars<br>*State explicitly what error bars represent (e.g. SD, SE, CI)*

Our web collection on statistics for biologists may be useful.

Software and code

Policy information about availability of computer code

Data collection	Microsoft Excel, ImageJ, Microsoft Powerpoint
Data analysis	Microsoft Excel, ImageJ, Microsoft Powerpoint, Plotly

For manuscripts utilizing custom algorithms or software that are central to the research but not yet described in published literature, software must be made available to editors/reviewers upon request. We strongly encourage code deposition in a community repository (e.g. GitHub). See the Nature Research guidelines for submitting code & software for further information.

Data

Policy information about availability of data

All manuscripts must include a data availability statement. This statement should provide the following information, where applicable:

- Accession codes, unique identifiers, or web links for publicly available datasets
- A list of figures that have associated raw data
- A description of any restrictions on data availability

No accession codes, unique identifiers, or web links for publicly available datasets. There are no restrictions on data availability. All figures have associated raw data other than main figure 4 and supplemental figures 3 and 4.

Field-specific reporting

Please select the best fit for your research. If you are not sure, read the appropriate sections before making your selection.

☒ Life sciences ☐ Behavioural & social sciences ☐ Ecological, evolutionary & environmental sciences

For a reference copy of the document with all sections, see nature.com/authors/policies/ReportingSummary-flat.pdf

Life sciences study design

All studies must disclose on these points even when the disclosure is negative.

Sample size	Sample sizes were chosen based on standards in the field and previously published literature. All tumor studies contained a minimum of 8 individual tumors for analysis, except for the PIP2 mouse tumor 'rescue' studies in which a minimum of 4 individual tumors were analyzed due to cost limitations.
Data exclusions	Abnormally developed fish were excluded from studies. Additionally, for the tumor studies done with allografts (implantations), any tumors whose size was two standard deviations or more outside of the mean for that treatment group were excluded from analysis. The two standard deviation cutoff protocol was established prior to any data generation, and was intended to account for potential errors in numbers of tumor cells injected. This protocol was applied uniformly to all treatment groups. Overall, no more than 5% of tumors were excluded from the study as a result.
Replication	All experiments have a minimum of three replicates to assure the consistency of the results. In the case of the mouse genetic tumor model, three different breeding pairs of mice generating the Cad5(PAC)-CreERT2;Cds2lox/lox mice were utilized for analysis.
Randomization	For tumor allograft studies using vMO's, LiCl, and L690-488 studies: mice were obtained and intermixed prior to starting the studies. Tumors were injected into the mice, then mice were selected randomly for use in different treatments with inhibitors.
Blinding	For tumor allograft studies using vMO's, LiCl, and L690-488 studies: the authors were not blinded during these studies, as the chemicals had to be injected daily and tumor growth measurements were taken at the same time. Two authors independently verified all tumor sizes. For the mouse EC inducible CDS2 KO genetic model: the authors were blinded to the genotype of the mice during all growth measurements. For data analysis and imaging of immunofluorescence: two rounds of measurements were made by two individual authors. One set was not blinded, while the second set of measurements were partially blinded- the author knew that measurements were being made from the same sample sets, but did not know what these sets were (eg, control vs. vivo MO treated tumors). Finally, all fish studies and cell culture studies were not blinded because of the overt phenotypes present. In most cases quantitative measurements were made to verify subjectively observable results.

Reporting for specific materials, systems and methods

Materials & experimental systems

n/a	Involved in the study	
☐	☒	Unique biological materials
☐	☒	Antibodies
☐	☒	Eukaryotic cell lines
☒	☐	Palaeontology
☐	☒	Animals and other organisms
☒	☐	Human research participants

Methods

n/a	Involved in the study	
☒	☐	ChIP-seq
☒	☐	Flow cytometry
☒	☐	MRI-based neuroimaging

Unique biological materials

Policy information about availability of materials

Obtaining unique materials	All materials utilized within this manuscript are readily available from the authors or commercial sources such as Sigma, Jackson Labs, ZIRC, KOMP Repository, AbCAM, Cell Signaling, and RD Systems

Antibodies

Antibodies used	p-Erk1/2, T. Erk, p-Akt, T. Akt (Cell Signaling Technologies), Tubulin, Caspase3 (Sigma), CDS2 (ProteinTech, 13175-1-AP); antibody product numbers are located in the methods section of the manuscript

Validation	All antibodies were validated by the manufactures.

Eukaryotic cell lines

Policy information about cell lines

Cell line source(s)	HUVEC- human umbilical vein endothelial cells (ATCC); B16-F10 mouse melanoma (JS Gutkind Lab); LLC mouse carcinoma (JS Gutkind Lab)
Authentication	the cell lines were not authenticated beyond the manufactures or lab's declaration
Mycoplasma contamination	cell lines were not tested for mycoplasma contamination
Commonly misidentified lines (See ICLAC register)	none

Animals and other organisms

Policy information about studies involving animals; ARRIVE guidelines recommended for reporting animal research

Laboratory animals	mus musculus, B6, males and females, less than 1.5 years of age; danio rerio, EK, males and females, embryonic to 5dpf
Wild animals	*Provide details on animals observed in or captured in the field; report species, sex and age where possible. Describe how animals were caught and transported and what happened to captive animals after the study (if killed, explain why and describe method; if released, say where and when) OR state that the study did not involve wild animals.*
Field-collected samples	*For laboratory work with field-collected samples, describe all relevant parameters such as housing, maintenance, temperature, photoperiod and end-of-experiment protocol OR state that the study did not involve samples collected from the field.*
